# Ecological correlates of chimpanzee termite fishing behavior in Mbam & Djerem National Park, Cameroon

**DOI:** 10.1002/ece3.70080

**Published:** 2024-07-24

**Authors:** Tyler C. Andres‐Bray, Jeffrey Smith, Ian Nichols, Ekwoge E. Abwe, Mary Katherine Gonder

**Affiliations:** ^1^ Department of Biology Drexel University Philadelphia Pennsylvania USA; ^2^ Cameroon Biodiversity Protection Plan Yaoundé Cameroon; ^3^ Department of Forest Resources and Environmental Conservation Virginia Tech Blacksburg Virginia USA; ^4^ Cameroon Biodiversity Association Douala Cameroon; ^5^ San Diego Zoo Wildlife Alliance San Diego California USA; ^6^ Department of Ecology and Conservation Biology Texas A&M University College Station Texas USA

**Keywords:** Nigeria‐Cameroon chimpanzee, seasonality, termite behavior, tool use

## Abstract

Chimpanzee insectivory is seasonally variable, with pronounced peaks or set seasonal periods of consumption observed in most chimpanzee communities. This variation is interesting given that chimpanzees invest considerable effort into complex tool‐using behaviors to acquire insect prey. Evidence suggests this seasonal variation is related to insect behavior, but few studies have been done to empirically examine this relationship. In this study, we assessed whether a seasonal pattern of termite fishing by Nigeria‐Cameroon chimpanzees (*Pan troglodytes ellioti*) in Mbam & Djerem National Park, Cameroon was driven by termite behavior. We measured termite presence and termite foraging activity monthly at seven termite mounds near Ganga Research Station from April 2022 to April 2023. Macroscopic fecal analysis and camera traps placed at each mound demonstrated termite fishing in this community occurred from March to June, with a rare smaller period of termite fishing in October 2021. Average monthly rainfall, average monthly temperature, and average monthly fruit availability were used to examine potential environmental factors that could impact termite fishing seasonality. Termite presence was significantly different between months with and without chimpanzee termite fishing (*t*‐test, −6.569, *p* < .001). Termite presence was also significantly associated with average monthly rainfall (ANOVA, *F* = 13.9, *p* = .002, *R* ^2^ = .775). Termites in this region appear to respond to the transition from dry to wet seasons by moving closer to the soil surface. This corresponds with greater chimpanzee termite fishing, suggesting that termite accessibility may be driving seasonal variation in this behavior.

## Graphical abstract

Using an interdisciplinary approach involving remote camera trap monitoring, termite behavioral surveys, and ecological surveys, we identified key ecological factors related to a pronounced seasonal period of termite fishing in a community of the understudied Nigeria‐Cameroon chimpanzee in central Cameroon. Our results suggest the highly seasonal nature of chimpanzee termite fishing in this region is driven by seasonal variation in termite behavior mediated by rain, which makes termites more accessible to chimpanzees during periods of intermediate rainfall.

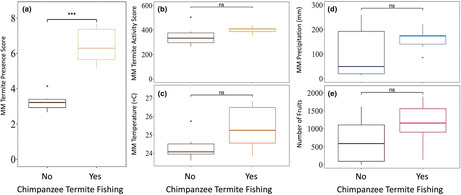

## 1 INTRODUCTION

Insects are an important dietary resource for many primate species (Isbell, 1998; MacKinnon & MacKinnon, 1980; Raubenheimer & Rothman, 2013). Despite insect prey being nutrient‐dense, obligately insectivorous primates tend to be small‐bodied (McGrew, 2014). Catching insect prey has high energetic costs relative to their energetic benefits, making it untenable for larger species to subsist solely on insect prey (Kay, 1975; McGrew, 2014). Some large‐bodied primates, like chimpanzees (*Pan troglodytes* spp.), overcome this limitation by predating upon social insects (e.g., termites and ants) with tools, which enables increased yield while reducing overall effort (Bogart & Pruetz, 2011; Goodall, 1964). These tool‐using behaviors are cognitively complex, have a prolonged learning period, and can vary substantially and culturally between communities (Boesch et al., 2020; Lonsdorf et al., 2004; Whiten et al., 1999). Insectivory, often via tool‐assisted insect consumption, is common across many chimpanzee communities, suggesting that insects play a vital nutritional role in chimpanzee diets, though the relative importance and the evolutionary value of this role remains an area of active research (Raubenheimer & Rothman, 2013; Whiten et al., 1999). While insectivory in chimpanzee communities is widespread, there is high inter‐community and seasonal variation in social insect consumption that suggests that the degree and type of social insect consumption in chimpanzees is influenced by ecological, biological, and/or social factors (Bogart & Pruetz, 2011; Dutton & Chapman, 2015; Stewart & Piel, 2014; Whiten, 2017). Examining the complex interplay between these factors and chimpanzee insectivory can help us understand how insect prey can potentially impact chimpanzee health and evolution.

Chimpanzee communities vary in the species, degree, and the seasonal periods in which they consume insects. Termites are eaten year‐round by chimpanzees at Fongoli in Senegal, Okorobiko in Equatorial Guinea, Gombe in Tanzania, and Goualougo in the Republic of the Congo (Bogart & Pruetz, 2011; McGrew et al., 1979; Sanz & Morgan, 2013). At Fongoli, the proportion of feeding time devoted to termites varies from nearly 0% to 60%, with the highest feeding times occurring at the transition from dry to wet seasons (Bogart & Pruetz, 2011). At Gombe, chimpanzees have been seen eating termites throughout the year, but there is a peak when the wet season begins (McGrew et al., 1979). However, at Okorobiko, there is little variation in consumption throughout the year (McGrew et al., 1979), termites are eaten throughout the year at Goualougo with the help of a unique sequential tool‐use behavior used to access deep subterranean termite nests (Sanz & Morgan, 2013). Conversely, consuming termites is highly seasonal in other chimpanzee communities, like Mt. Assirik in Senegal and Issa Valley in Tanzania. At Mt. Assirik, tools used to acquire termites are only found from May to September, with 89% of these tools found specifically in June (McGrew et al., 1979). In the Issa Valley, chimpanzees only eat termites at the start and end of the wet season, comprising approximately 6 months of the year (Stewart & Piel, 2014). Similar seasonal patterns of consumption have been seen with ants. At Mahale in Tanzania, chimpanzees eat ants year‐round, but ant predation decreases in the late wet season, corresponding to an increase in termite predation (Nishida & Hiraiwa, 1982). Both ants and termites are considered important staple foods for chimpanzees at La Belgique in southern Cameroon, and they are eaten throughout the year (Deblauwe, 2009).

To date, empirical research into consumption of social insects has been limited for the Nigeria‐Cameroon chimpanzee subspecies (*Pan troglodytes ellioti*). Chimpanzees have not been seen preying upon termites at either Gashaka or Ngel Nyaki in Nigeria, nor have any tools associated with acquiring termites been seen despite both sites having termite mounds present (Dutton & Chapman, 2015; Sommer et al., 2017). However, both sites have evidence of predation on ants, with army ant consumption at Gashaka remaining high and consistent throughout the year, while chimpanzees at Ngel Nyaki show evidence of ant consumption more periodically throughout the year (Dutton & Chapman, 2015; Sommer et al., 2017). Finally, chimpanzees at Ebo Forest in western Cameroon eat termites and ants variably throughout the year (Abwe, 2018; Abwe & Morgan, 2008).

Several nonmutually exclusive hypotheses have been suggested that involve ecological factors to explain the origin and maintenance of tool use in hominids (Fox et al., 1999). The “necessity hypothesis” argues that tool use is driven by food scarcity, and tools enabled great apes to access high‐value embedded resources such as social insects and nuts (Fox et al., 1999). This hypothesis has some support in savanna environments where termites are eaten year‐round (e.g., Fongoli), though rates of termite consumption were not correlated with fruit availability (Bogart & Pruetz, 2011; Hernandez‐Aguilar et al., 2007). Low resource density in these areas compared to more stable rainforest habitats may promote the consumption of any viable food items when they are available, and Fongoli has a high density of termites (Bogart & Pruetz, 2011; Hernandez‐Aguilar et al., 2007). A second hypothesis, the “opportunity hypothesis”, argues that tool use has emerged and is shaped from repeated exposure to appropriate conditions promoting tool use (e.g., insect prey characteristics, tool material availability, etc.) (Fox et al., 1999). Evidence for this hypothesis includes seasonal variation in tool use that is unrelated to the availability of preferred foods, such as at Gombe, where peak termite feeding occurs at the start of the wet season when fruits are abundant (McGrew et al., 1979), and at Goualougo, where their sequential tool use allows access to termites in deep nests year‐round (Sanz & Morgan, 2013).

Strong seasonality of certain categories of tool use due to ecological constraints imparted by climate factors or prey behavior would support the “opportunity” hypothesis. One recent study by Phillips et al. (2023) found that termite fishing seasonality in a chimpanzee community in Issa Valley, Tanzania, was shaped in part by termite accessibility relating to termite reproductive behavior. Researchers observed that rainfall, humidity, and wind speed in this region were associated with dispersal flights of reproductive *Macrotermes* alates and that this behavior was accompanied by increased termite abundance in mound flight holes that made them more vulnerable to researcher‐based termite fishing experiments (Phillips et al., 2023). In this study, we examined seasonal fluctuations in termite fishing, a well‐documented chimpanzee tool‐using behavior where a plant probe is inserted into termite mounds to extract termites (Boesch et al., 2020; Goodall, 1964), in a community of *P. t. ellioti* in Mbam & Djerem National Park (MDNP) in central Cameroon. We sought to determine if this seasonality fulfills the “opportunity” and/or the “necessity” hypotheses. Previous research has found that chimpanzees in this community rely more on animal matter in their diets relative to chimpanzees in west Cameroon (Abwe, 2018). Specifically, we seek to address the following questions:

1. Is seasonal variation of termite fishing in MDNP related to seasonal variation in termite behavior (opportunity) or fruit availability (necessity)?
2. What environmental factors drive seasonal variation in termite behavior in MDNP?

The findings of Phillips et al. (2023) suggest that chimpanzees may be taking advantage of increased termite activity around reproductive periods and that termites may be otherwise inaccessible to chimpanzees during other parts of the year. In this study, we tested this possibility in an unhabituated community of *P. t. ellioti* in MDNP where anecdotal evidence has revealed a highly seasonal period of termite fishing.

## 2 METHODS

### 2.1 Study site

**FIGURE 1:**
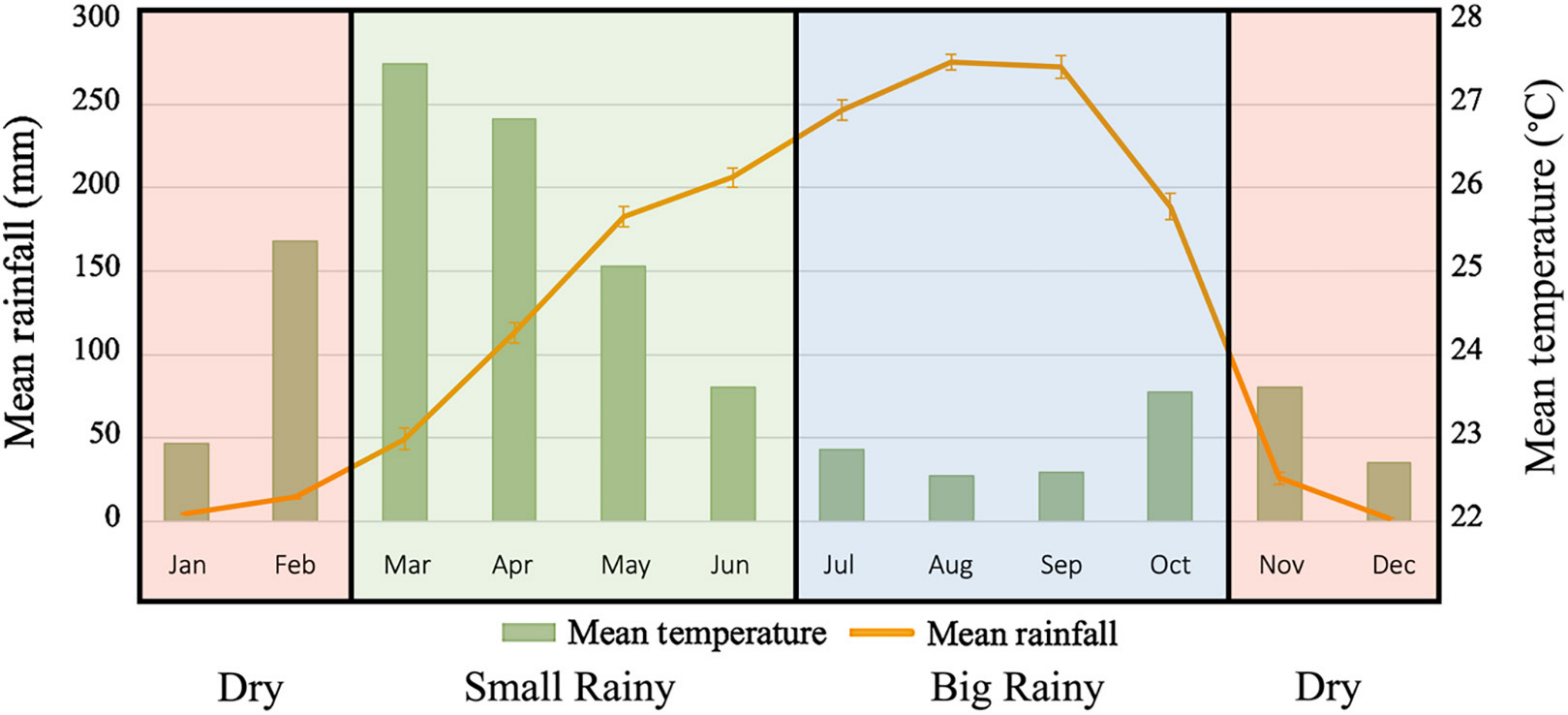
Seasonal breakdown of Mbam & Djerem National Park based on rainfall and temperature. The dry season occurs generally from approximately November to February. The wet season can be split into the small rainy season from March to June, and the big rainy season from July to October. The small rainy season is a transitionary season marked by steadily increasing rainfall and high average temperatures, while the big rainy season has high average rainfall and low average temperatures. Error bars for rainfall line represent standard error. Data for this figure were acquired from 1991 to 2020 from the Climate Research Institute.

This study was conducted at Ganga Research Station in Mbam & Djerem National Park (MDNP). MDNP is a 4152 km^2^ protected area located in central Cameroon in a complex forest‐woodland‐savanna ecotone habitat at the conjunction of the Guinean rainforest, Congolian rainforest, and Sahel savanna biomes (Maisels, 2005; Smith et al., 1997). It receives an annual rainfall of approximately 2000 mm, with a dry season from roughly November to February, and a wet season from March to October (Abwe, 2018). This wet season can be further divided into a small rainy season from March to June when temperatures are high and rainfall is beginning to increase, and a big rainy season from July to October when temperatures are lower and rainfall is higher (Figure 1). MDNP is an important refuge for the Endangered *P. t. ellioti*, as previous research has found that the park contains over 500 individuals at a density of 0.33 chimpanzees per km^−2^ (0.12–0.86 CI) (Kamgang et al., 2018; Oates et al., 2016). Ganga Research Station, where this study took place, is a remote site in the northeastern portion of MDNP and is only accessible via boat on the Djerem River (Abwe, 2018).

### 2.2 Data collection

#### 2.2.1 Determining termite fishing seasonality

**FIGURE 2:**
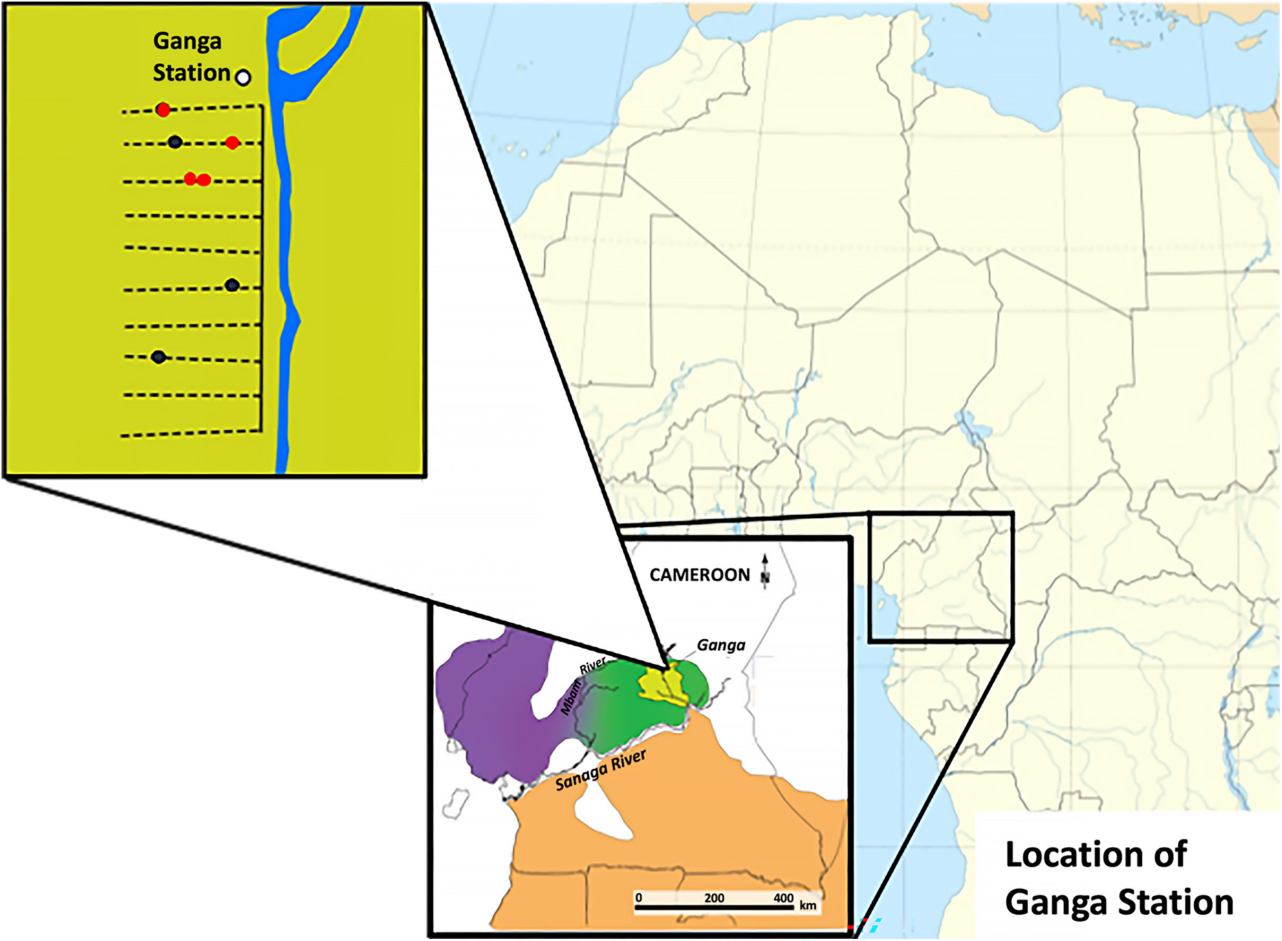
Location of termite mounds along transects near Ganga Research Station: Ganga Research Station is found in the northeastern part of Mbam & Djerem National Park, which is located in both the Adamawa and Centre regions of central Cameroon. Since 2016, monthly biomonitoring has been conducted along 10 2‐km‐long transects that extend perpendicular to the Djerem river at 0.5 km intervals. The red and black dots represent termite mounds where camera traps were placed. Red dots are mounds where chimpanzees have been seen on camera termite fishing while black dots are mounds where we captured no video evidence of chimpanzee termite fishing. Termite mounds where chimpanzee termite fishing was observed are assumed to be Sphaerotermes sphaerothorax based on comparative abundances of termite specimen identified via morphological characteristics, though this is not definitive (See Section 2.2.2 for further information). Purple, green, and orange areas in the map represent genetically distinct populations of chimpanzees from Mitchell et al. (2015): Two *P. t. ellioti* populations in a rainforest habitat (purple) and ecotone habitat (green), and one population of *P. t. troglodytes* (orange).

We identified months when chimpanzees participated in termite fishing using a combination of direct evidence from videos and indirect evidence from termite fishing tool collection and macroscopic diet analysis from fecal samples. Motion‐activated Browning Spec Ops Advantage camera traps were stationed at all termite mounds identified along 10 transects near Ganga Research Station (*n* = 7) in Mbam & Djerem National Park, Cameroon (Figure 2) from 2020 to 2023. These cameras were set to record 1‐min videos (20 s in low‐light settings) whenever they detected motion. Field biologists working for the Cameroon Biodiversity Protection Program and Cameroon Biodiversity Association collected SD cards and replaced batteries for each camera monthly, and videos were uploaded to a shared Dropbox folder. We cataloged each video based on the presence of animals, animal species present, and the occurrence of chimpanzee tool use. The seven mounds in this study have an mean inter‐mound distance of ~1596 m based on their locations along each transect and the distance between each transect, with the most distant mound (Mound A on Transect 8) located ~1720 m from the next nearest termite mound, and with mounds B and E located nearest to one another at a distance of ~50 m down the same transect (Figure 2).

Additionally, field biologists collected chimpanzee tools (2019–2022) and chimpanzee fecal samples (2016–2017) opportunistically during monthly biomonitoring patrols. When possible, these field biologists noted the species, dimensions, and prey targets for each chimpanzee tool collected. Insect prey target was determined from previous knowledge of social insect colonies at each location (e.g., when tools were collected adjacent to a known termite mound, tools were cataloged as termite fishing tools) or from active searching for insect nests in unfamiliar locations. Tools where the prey target could not be readily identified were not used in this study. For chimpanzee fecal samples, the field biologists would wash the fecal sample in camp to reveal diet items and would quantify the proportion of fruit, fiber, and animal matter for each sample. When possible, the species of all fruit and animal matter were also identified.

**FIGURE 3:**
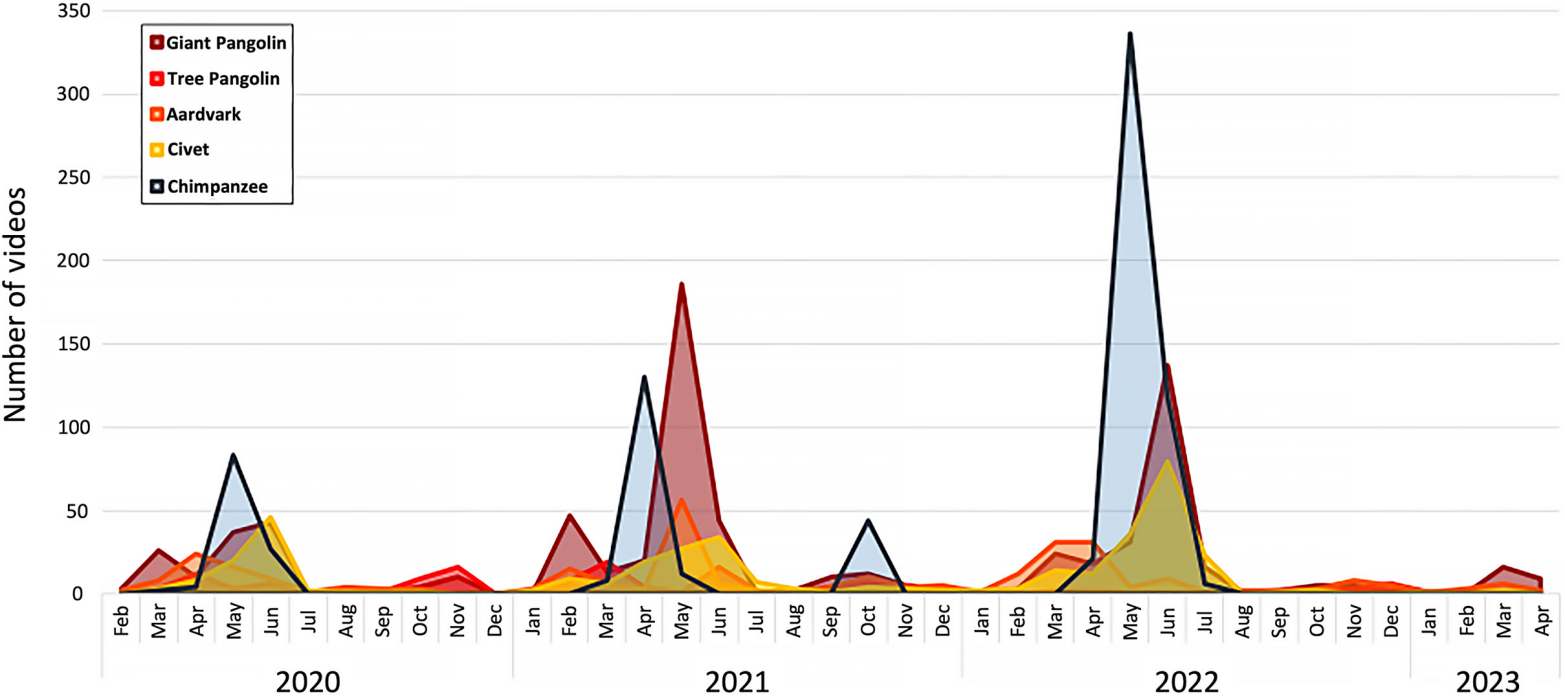
Temporal variation in consumption of mound‐building termites near Ganga Research Station. This graph shows the number of camera trap videos depicting termite consumption per month of chimpanzees (*Pan troglodytes ellioti*) as well as several known termite specialists: (1) Giant pangolin (*Smutsia gigantea*), (2) Tree pangolin (*Phataginus tricuspis*), (3) Aardvark (*Orycteropus afer*), and (4) African civet (*Civettictis civetta*). This demonstrates a pattern across all of these species of prolonged termite consumption in the early half of the year with a spike in activity around March–June, and a smaller secondary period of termite consumption around October/November that is seen primarily in the termite specialists. It is important to note that all videos of chimpanzee termite fishing that occurred in October during the 3‐year course of this project were a single fishing event by a single chimpanzee.

Camera trap videos captured instances of termite fishing from April to June during the project, with one instance of a single chimpanzee fishing during October 2021. This pattern is shared by other mammals in this region that are termite specialists (e.g., pangolins, aardvarks, etc.), where there is a high degree of termite consumption from March to June, and a second smaller period of termite consumption in October and November (Figure 3). Figure 3 uses number of videos as a broad measure of activity at the termite mounds by chimpanzees and other termite‐specialized mammals, as these videos capture visits of several seconds to visits of several hours captured across a number of sequential videos, and many of the species recorded cannot be identified by individual to provide an accurate account of the number of individual visitations. Termite tools were only found from May to July (May: *N* = 31, June: *N* = 33, July: *N* = 3), and termites identified in chimpanzee fecal samples from March to June (March: 3.13% of samples, April: 28.57% of samples, May: 10.00% of samples, June: 6.25% of samples). This indicates that termite fishing in the Ganga chimpanzee community has a strong seasonal signal from March to July, with strong peaks from April to June and a potential secondary period in October. It should be noted that because data collection by biomonitors occurs once per month, and this occurs between approximately the 8th and 18th day of the month, tools collected may represent tools used by chimpanzees in the previous month. However, to ensure our analyses are conservatively covering the extent of the termite fishing periods in this community, we use March to July plus October as months when chimpanzees are potentially termite fishing at Ganga.

#### 2.2.2 Termite mound survey

**FIGURE 4:**
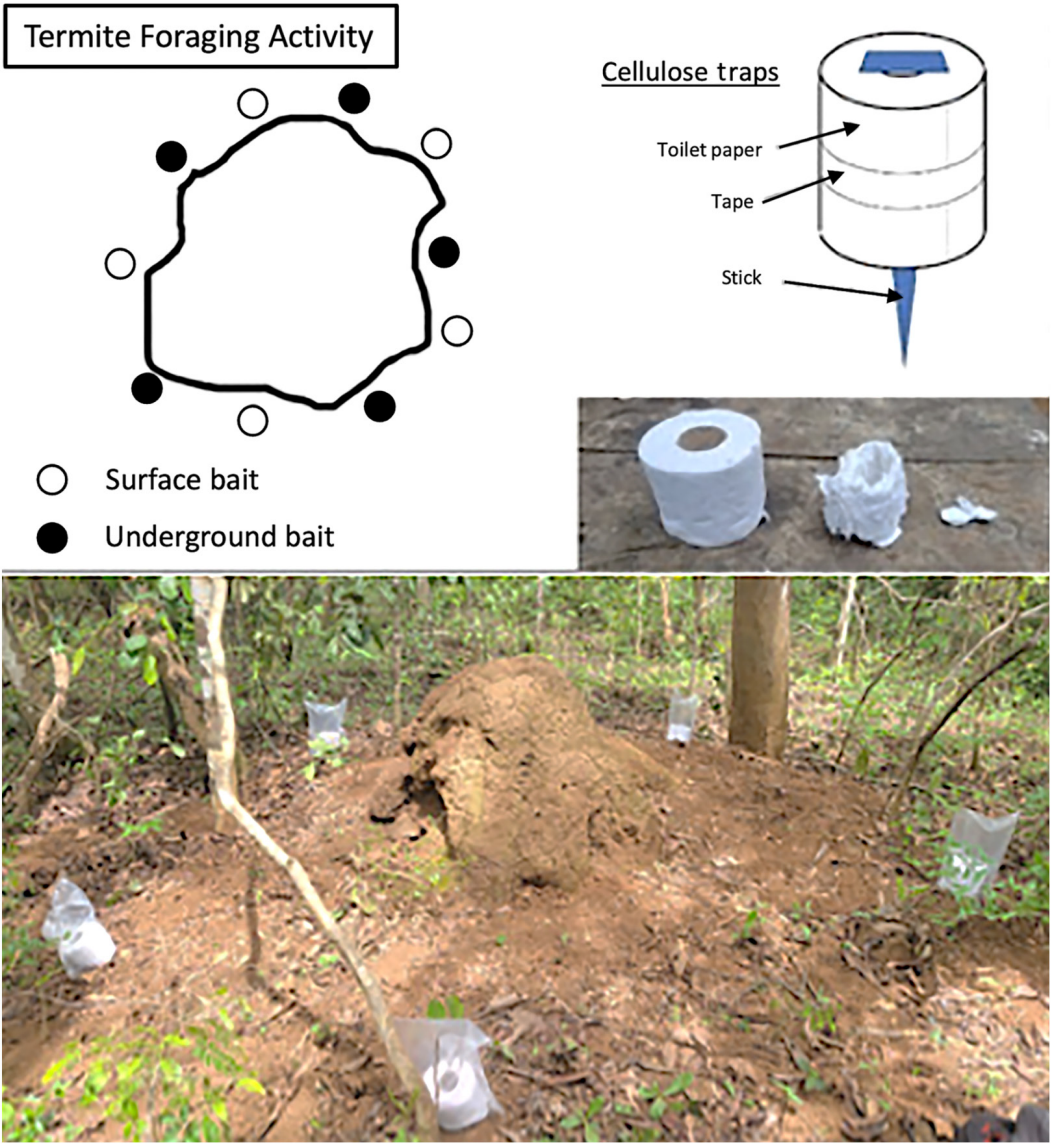
Diagram of termite foraging activity data collection. Termite foraging activity was measured monthly using 10 cellulose baits composed of toilet paper, with half being buried underground and half being secured to the soil surface using a modified stick. Together, these baits encircled each mound and were replaced monthly. Baits secured to the surface were covered with plastic bags to prevent wear from environmental factors. The above photograph includes examples of baits at various levels of consumption and an example set‐up at one of the termite mounds surveyed in this study.

Termite foraging activity was quantified monthly from April 2022 to April 2023 using cellulose baits (in the form of 500‐sheet unscented toilet paper rolls) placed around each of the preidentified seven termite mounds near Ganga Research Station. Cellulose baits have been used in the past to measure activity patterns and species diversity of xylophagous termites (Davies et al., 2015). Each termite mound had 10 baits, five secured to the soil surface, and five buried 5 cm below the soil surface in an alternating pattern (Figure 4). The cellulose baits secured to the soil surface were staked down with sharpened sticks collected near each mound to minimize the number of man‐made implements used in the survey. Each surface bait was also covered with a plastic bag to reduce wear from weather and larger animals. Each month, all cellulose baits were collected, assessed for termite activity, and replaced. All cellulose baits were visually graded by field biologists D. Syno, N Djimenet, and J Wanmetching with an ordinal 0–5 activity score to represent the amount of bait that had been consumed (0 = 0%, 1 = 1%–25%, 2 = 26%–50%, 3 = 51%–75%, 4 = 76%–99%, 5 = 100%) (Davies et al., 2015). All three field biologists went through an initial training period to ensure their bait grades would align, and baits were always scored by at least two field biologists. Baits would not be scored if there was evidence of disruption done by other, larger organisms (e.g., chimpanzees).

Termite specimens from the cellulose baits were used to attempt to identify termite species at each mound. A number of termite species were found in baits at each mound. At the four mounds where chimpanzee termite fishing was seen, termite communities were dominated by *Nasutitermes* spp. (31.3% of specimens), *Sphaerotermes sphaerothorax* (23% of specimens), and *Microtermes calvus* (11.3% of specimens). Members of *Nasutitermes* frequently build arboreal nests, suggesting that they are not the species being fished by chimpanzees (Thorne et al., 1996). *S. sphaerothorax* and *M. calvus* are both in the subfamily Macrotermitinae, although *S. sphaerothorax* is a unique member as they do not cultivate fungus in the combs of their nest as do other members of Macrotermitinae (Darlington, 2021). While comparably little is known of *S. sphaerothorax* behavior, they have been observed in epigeal nests, while *Microtermes* spp. are often subterranean but known to coexist with mound‐building termites (Darlington, 2021; Wood, 1988). Based on this information, termites fished by chimpanzees in this study are assumed to be *S. sphaerothorax*, but this is not known for certain, and we further cannot exclude the possibility that at least some of the mounds being fished are inhabited by *M. calvus*. Termites belonging to the *Macrotermes* genus, which are a known target of chimpanzee termite fishing tool use in multiple communities (McGrew, 2014), were identified at several mounds in very small abundances (1.7% of specimens).

**FIGURE 5:**
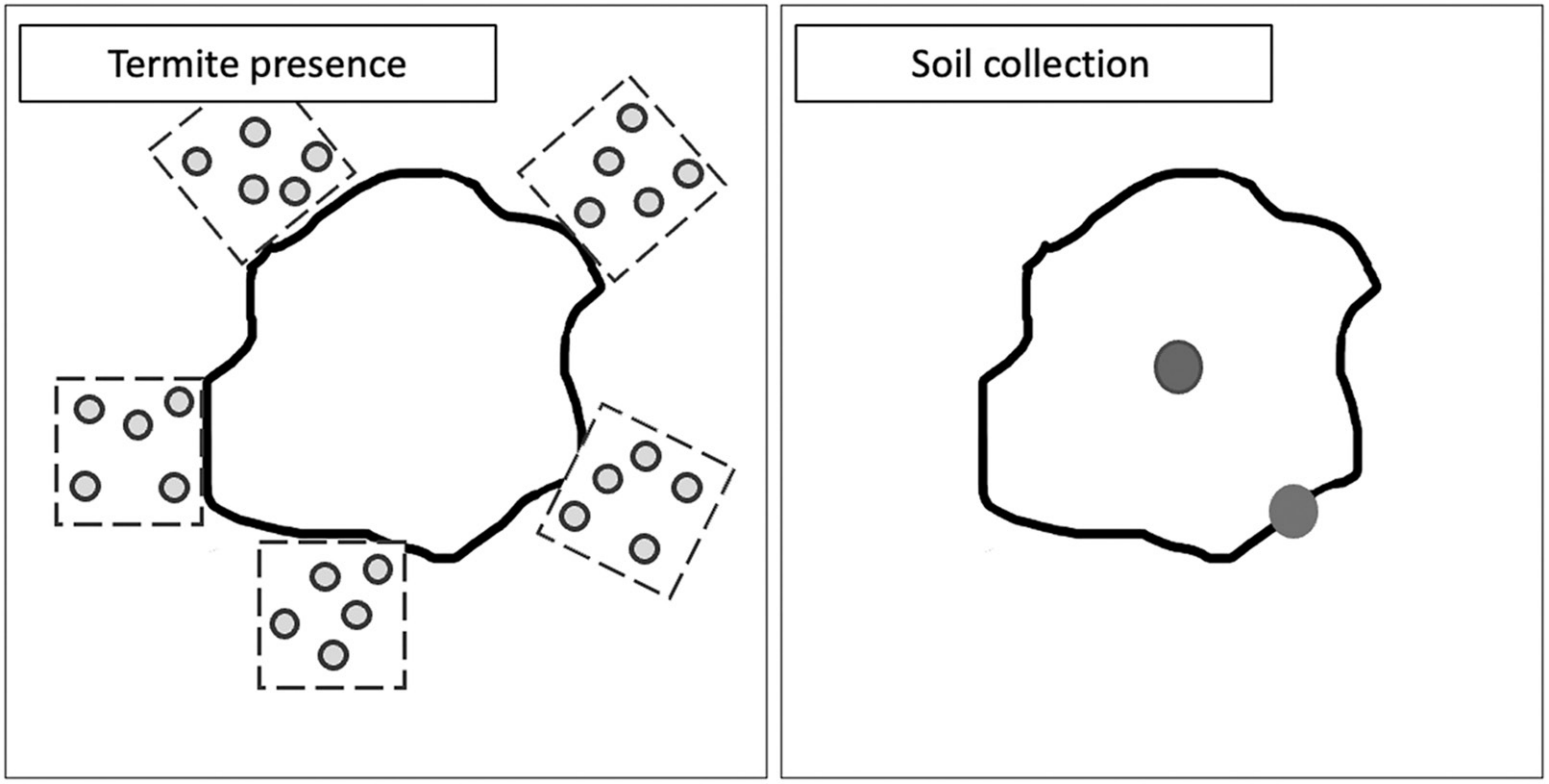
Diagram of termite presence data collection and soil sample collection. Termite presence in the soil surface was measured through active searching in five 1 m × 1 m quadrats tangent to each termite mound. Five 6‐inch‐deep holes were dug in each quadrat (light gray circles) and the level of termite presence was quantified for per quadrat. Every two months, two soil samples were collected from each mound (dark gray circles), one from the apex and one from the base.

During monthly bait replacements, field biologists conducted active searching for termites in five 1 m^2^ square quadrats tangent to each termite mound (Figure 5). Five 6‐inch‐deep holes were dug in each quadrat at random and the number of termites present were quantified using an ordinal 0–5 scale (0 = no termites, 1 = 1–25 termites, 2 = 26–50 termites, 3 = 51–75 termites, 4 = 76–100 termites, 5 = more than 100 termites). An ordinal scale was chosen to facilitate quick and efficient assessments of termite presence given the large amount of sampling (*N* = 175 per sampling) that would only be possible at this site for approximately 1 week every month due to the site's remote location in MDNP.

Two soil samples were collected from each termite mound bimonthly to explore inter‐mound variation and seasonal variation in soil quality. One sample was taken from the apex of the termite mound, and one was taken from the base (Figure 5). If the termite mound was an underground nest with minimal above‐ground structure, one sample was taken from the center and one was taken from the edge. These samples were transported to the Cameroon Biodiversity Protection Program office in Yaoundé, where Y. Djouguela would assess soil pH and water content. Soil pH was analyzed by using an OHAUS™ Starter pH Pen Meter on a 2:5 ratio soil‐to‐water mixture with a portion of each soil sample. Water content was measured using a FEEDRON Digital Milligram Scale. Soil from each sample was weighed and subsequently dried using silica. The dried soil was reweighed, and water content was calculated as 1 – Weight_dry_/Weight_original_, thus representing the proportion of the soil weight attributable to water.

#### 2.2.3 Ecological data collection

Monthly temperature and monthly rainfall were averaged across 1991 to 2020 using data obtained from the Climate Research Unit (World Bank Group, 2020). As the Climate Research Unit is able to show climate data down to the regional scale, we used data from the Adamawa region of Cameroon, as Ganga Research Station falls within this region.

Fruit availability was assessed based on monthly transect surveys that occurred between January 2016 and September 2017, and from January 2019 through December 2021 (Abwe et al., 2019). All fallen fruits known to be chimpanzee dietary items were identified and counted on a 1 m band along each transect (Figure 2) (Furuichi et al., 2001). We calculated the average monthly fruit availability based on the combined monthly fruitfall of the 10 most preferred chimpanzee fruits in this community (*Landolphia* spp., *Uapaca guineensis*, *Ficus* spp., *Pseudospondias macrocarpa*, *Myrianthus arboreus*, *Grewia* spp., *Canarium schweinfurthii*, and three unidentified fruit species for which we obtained seeds for reference). These were determined to be preferred based on macroscopic analysis of chimpanzee feces for fruit seeds, and these 10 fruits were the most prevalent as well as the only fruits that made up more than 50% of monthly chimpanzee fruit consumption for at least 1 month (Abwe, 2018).

### 2.3 Data analysis

#### 2.3.1 Preparation of termite data

**FIGURE 6:**
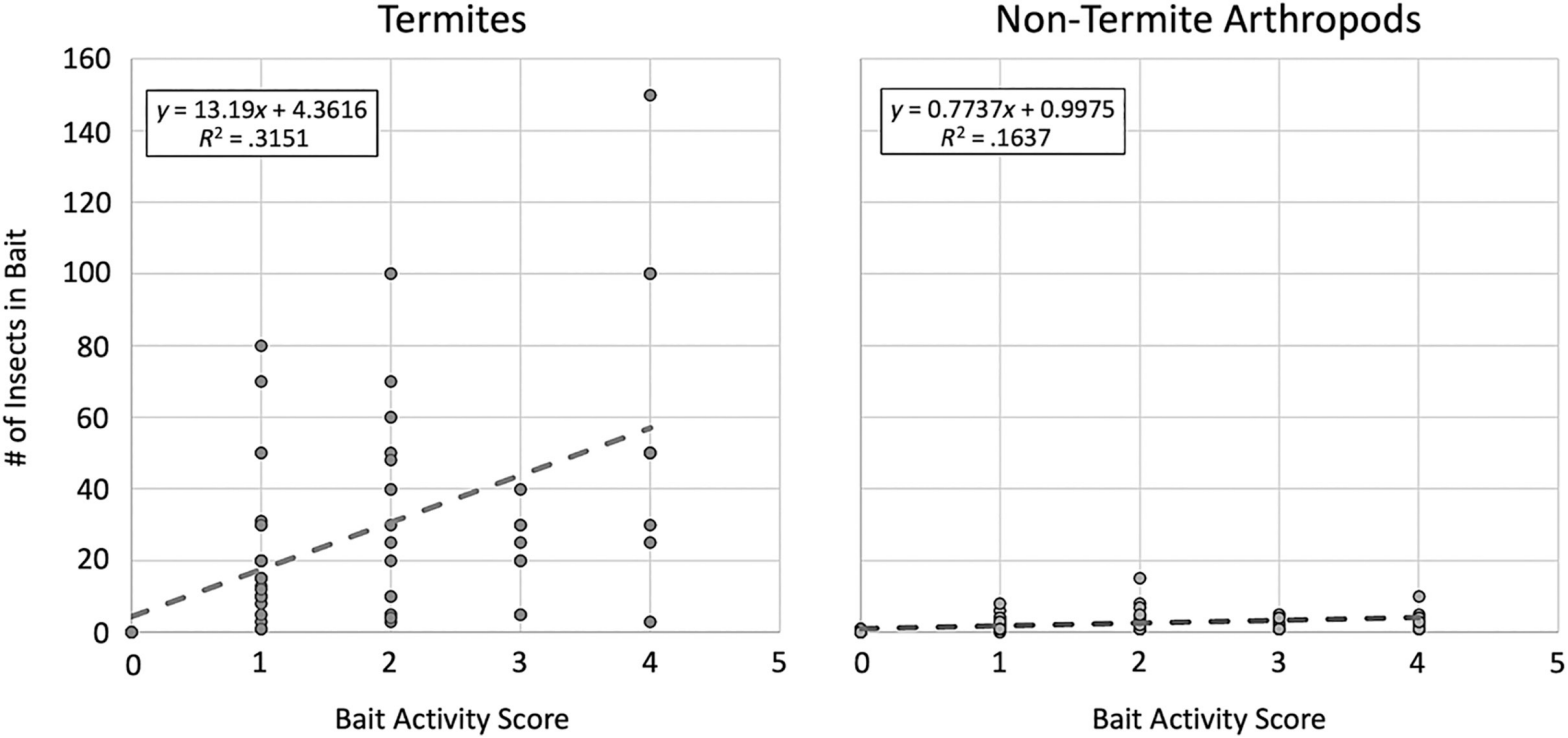
Assessment of cellulose bait scores. The figure on the left shows the relationship between number of termites found per bait during collection and the activity score for that bait. The figure on the right shows the same information for all nontermite arthropods found in each bait. Overall, far more termites were seen per bait than other arthropods, and the number of termites in each bait had a better goodness of fit with the bait score compared to nontermite arthropods (Termites: *R* ^2^ = .315, Nontermite Arthropods: *R* ^2^ = .164), suggesting that termites are more responsible for bait consumption than other insects.

During the assessment of cellulose baits, the field biologists counted the number of termites and nontermite arthropods present in the baits. The quantification of termite and nontermite arthropods was used to ensure that the activity score was measuring termite activity. We used linear regression to compare the number of termites and the number of nontermite arthropods with the bait score and found that the number of termites present in the baits was more strongly related to activity score than nontermite arthropods (Figure 6). We excluded baits that were completely consumed (activity score = 5), because there may be no termites present at the location of this bait if the entire bait was finished days or weeks before replacement. Furthermore, because each mound was only visited once per month, there was high variation in the number of termites within each activity score that could be the result of the time of day the mound was visited, or how long it had been since the termites had found the bait. For this reason, we used the equation generated from the linear regression between activity score and number of termites found in the baits to transform the activity score. This would conservatively adjust the activity score to more accurately reflect only foraging activity performed on the cellulose baits by termites.

Transformed activity scores and termite presence scores were both summed per mound for each month, as summation of ordinal data can allow ordinal values to approximate continuous values. These summed activity scores and presence scores were then assessed for normality and averaged per month to create a monthly termite activity value and a monthly termite presence value which were used in subsequent analyses.

#### 2.3.2 Statistics

Shapiro–Wilk normality tests were used to confirm that the termite variables (mean monthly termite presence in the soil surface and mean monthly termite activity) and ecological variables (mean monthly temperature, mean monthly rainfall, and mean monthly preferred fruit availability) measured were normally distributed (Hanusz et al., 2016). We then employed two‐sided *t*‐tests for each termite and ecological variable to determine if their averages were different between months where chimpanzees did and did not participate in termite fishing. We then used one‐way ANOVA to determine if these variables differed between the three regional seasons (dry, small rainy, and big rainy). Finally, we used multiple regression to examine how rainfall and temperature impacted the termite variables to see if seasonality of termite behavior was mediated by environmental factors.

## 3 RESULTS

### 3.1 Ecological data

#### 3.1.1 Temperature and rainfall patterns

Temperature and rainfall variation in this region support the division of seasons used in our analyses. Mean monthly temperatures were lowest during the big rainy season (November to February) and highest during the small rainy season (March to June). Results of a one‐way ANOVA show this difference was significant (*F* = 7.344, *p* = .013). Mean monthly rainfall was lowest during the dry season and highest during the big rainy season (July to October). This difference was also significant based on a one‐way ANOVA (*F* = 28.51, *p* < .001). Together, this demonstrates that the monthly division we employed in this study is a valid representation of climatically distinct seasonal periods.

#### 3.1.2 Seasonal fruit availability of chimpanzee preferred fruits

**FIGURE 7:**
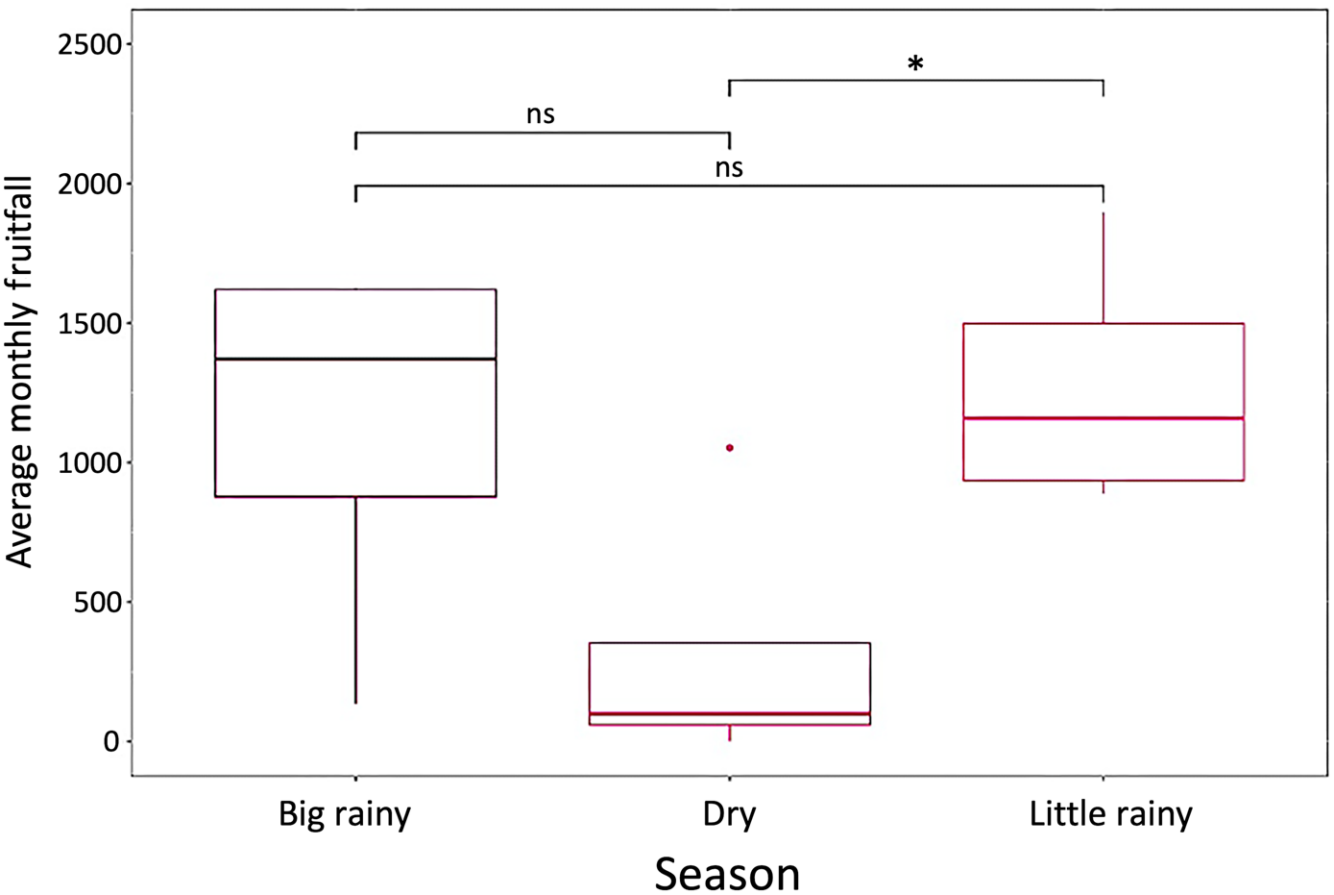
Fruit availability distribution between seasons. This figure shows the average monthly fruitfall of preferred chimpanzee fruits at Ganga Research Station using biomonitoring data from 2016 to 2021. The average monthly fruitfall was determined using the mean of each month's number of fallen fruits observed along the 10 2 km transects near Ganga Station. Preferred fruits represent the 10 most commonly found fruit species in chimpanzee fecal samples in this area. Preferred fruits are more available in the small rainy season (March to June) and the big rainy season (July to October) compared to the dry season (November to February) (*F* = 3.378, *p* = .081). Significance values represent the following: *p < .05, **p < .01, ***p < .001, ns = non‐significant.

Preferred chimpanzee fruits in this community appear to become available during the small rainy season, and overall preferred fruit availability remained stable through the big rainy season (Figure 7). These fruits were less abundant during the dry season, and this difference was near significant relative to the two rainy seasons (*F* = 3.378, *p* = .081).

#### 3.1.3 Seasonal variation in termite behavior

**FIGURE 8:**
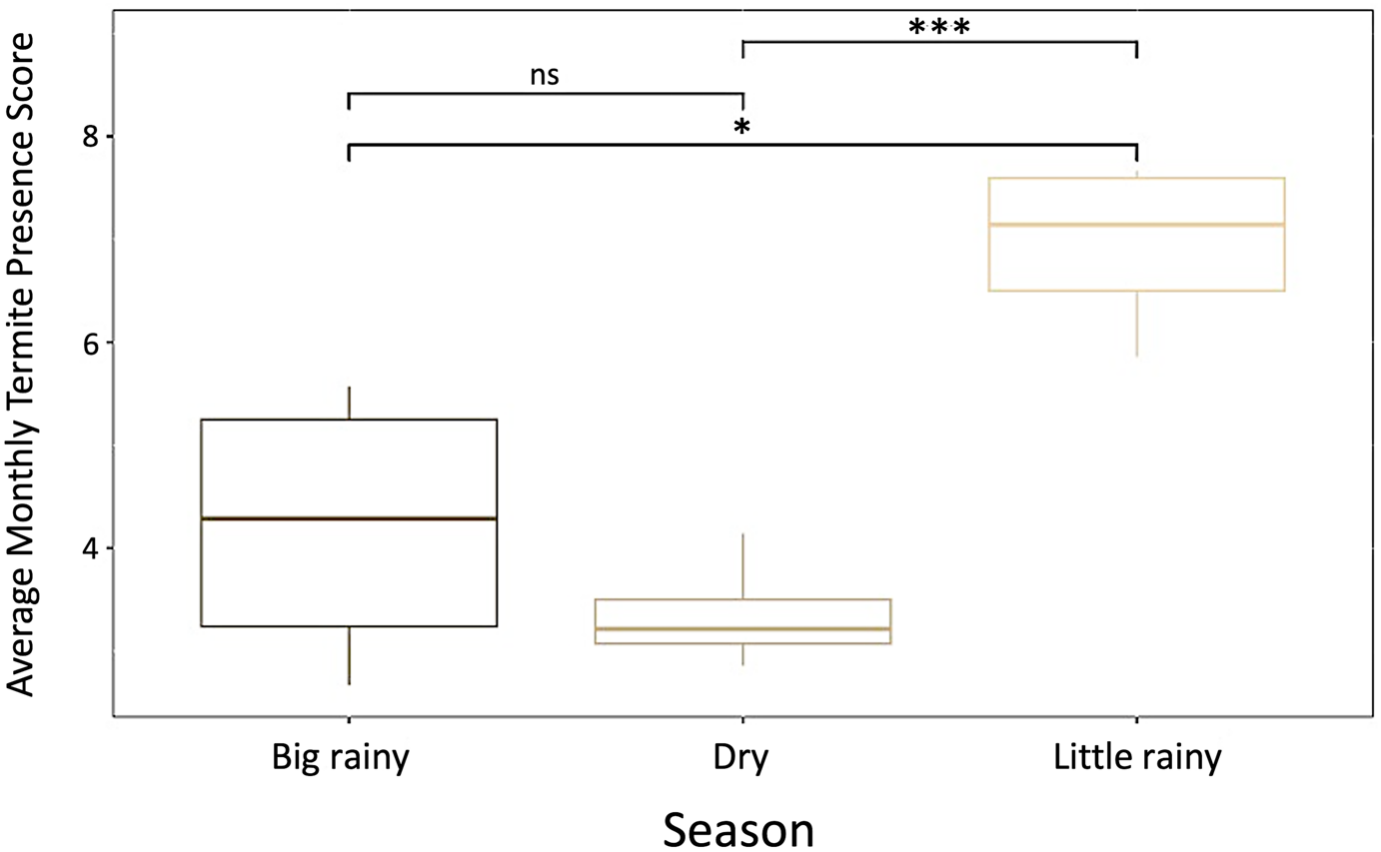
This figure shows the average monthly termite presence score per season from April 2022 to March 2023. The average monthly termite presence score was calculated by taking the sum of termite presence scores from each quadrat and calculating the mean of those summed scores each month across all seven termite mounds. Termite presence in the soil surface was significantly greater during the small rainy season (March–June) compared to the other two seasons (*F* = 14.48, *p* = .002). Significance values represent the following: *p < .05, **p < .01, ***p < .001, ns = non‐significant.

**FIGURE 9:**
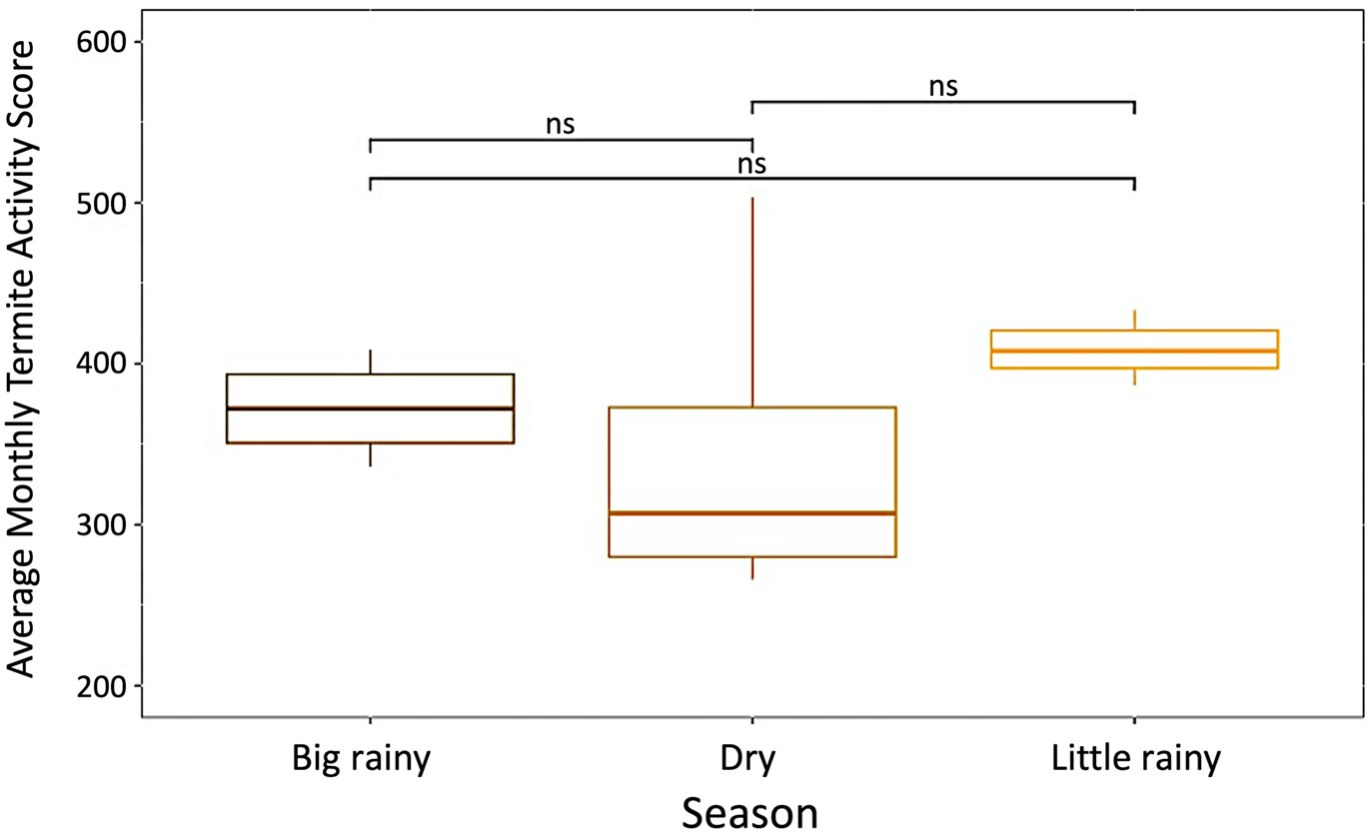
Termite foraging activity score distribution between seasons. This figure shows the average monthly termite activity score per season from April 2022 to March 2023. The average monthly termite activity score was calculated by calculating a corrected termite activity score for each termite bait using a regression equation relating the assigned bait score with the number of termites found per bait. These corrected scores were then summed across all baits per mound and then we calculated the mean of these summed corrected scores each month across all seven termite mounds. Termite foraging activity was not significantly different between each season (*F* = 0.699, *p* = .525). Significance values represent the following: *p < .05, **p < .01, ***p < .001, ns = non‐significant.

Termite presence in the upper soil surface was significantly higher during the small rainy season relative to the big rainy and dry seasons (*F* = 14.48, *p* = .002) (Figure 8). This increased presence in the upper region of the soil may be associated with mound‐building termite reproductive behavior, as anecdotal evidence from field biologists with the Cameroon Biodiversity Protection Program suggests the mound‐building alates in this region have been made their reproductive flights around May. Additionally, termite presence in the soil surface was consistently lowest in the dry season, which may be the result of termites traveling lower in their subterranean nests during this period in response to desiccation pressure (Woon et al., 2019). Termite foraging activity was not significantly different between seasons (*F* = 0.699, *p* = .525) (Figure 9). Termite foraging activity scores showed greater variation between mounds during the dry season, which may be related to termites balancing a need to forage for food/plant matter with the need to avoid desiccation (Figure 9). Together, these results suggest that there is seasonal variation in termite behavior related to their presence in the soil surface, but foraging behavior remains consistent throughout the year.

#### 3.1.4 Seasonal variation in termite mound characteristics

**FIGURE 10:**
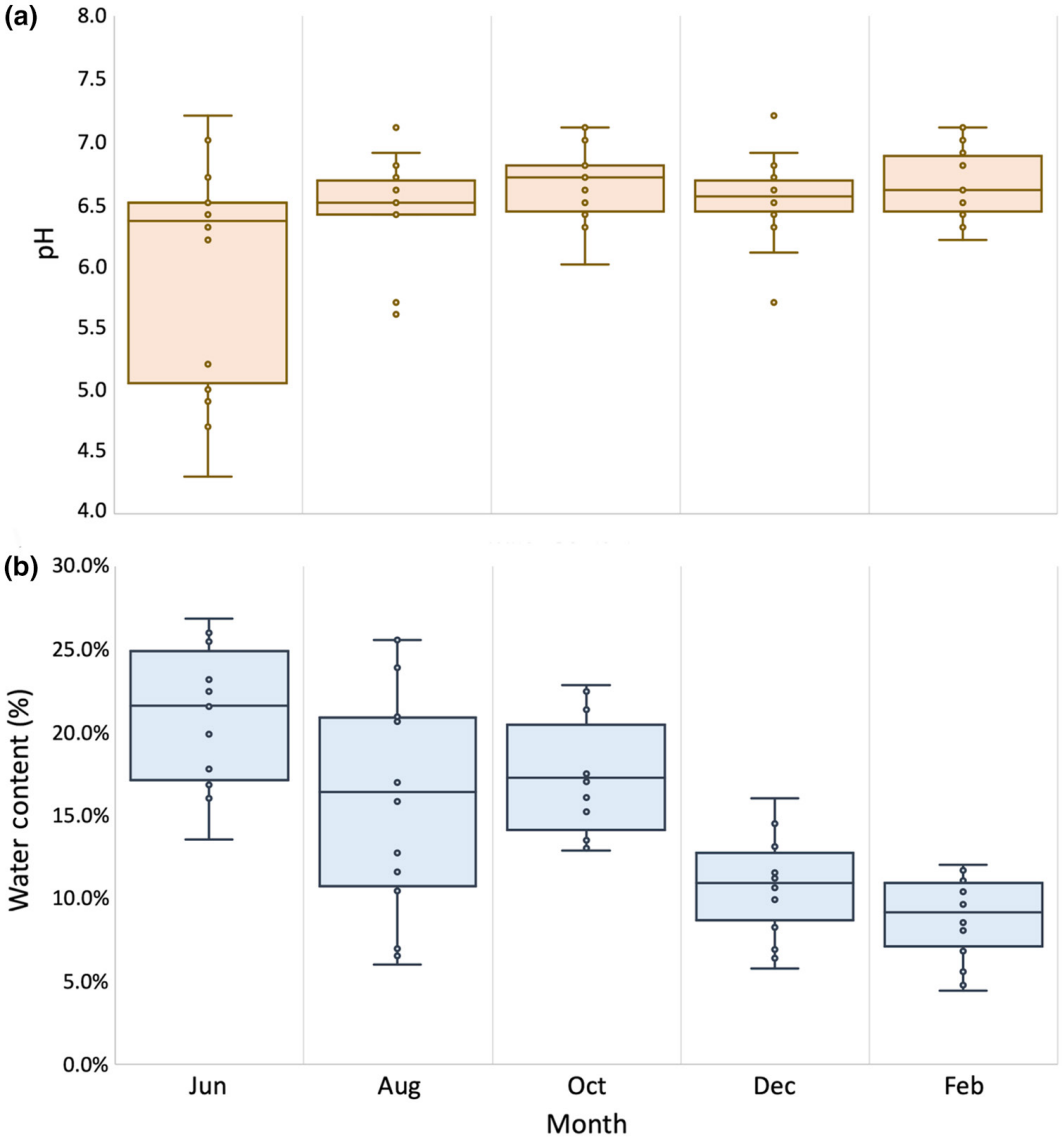
Bimonthly variation in pH and water content of termite mound soil. These figures show the distribution monthly values of mound soil pH (a) and water content (b) from June 2022 to February 2023 for the seven termite mounds monitored during this project. The pH of the termite mound soil remained relatively consistent throughout the year with a slightly acidic mean value of approximately 6.5. Water content was more variable, with the highest water content seen in June at the transition from the small rainy season to the big rainy season, and the lowest water content was observed in February during the dry season.

Median water content and pH values of bimonthly soil samples across all seven termite mounds demonstrate that pH remained predominantly consistent throughout the year, while water content varied (Figure 10). Median water content was highest in June (21% of sample) at the transition between small rainy and big rainy seasons, which also corresponds to the period of highest termite presence in the soil surface (Figure 10b). The median water content proceeded to decrease in subsequent months until reaching the lowest during February (8.8% of sample) in the dry season.

#### 3.1.5 Ecological correlates of termite fishing seasonality

**FIGURE 11:**
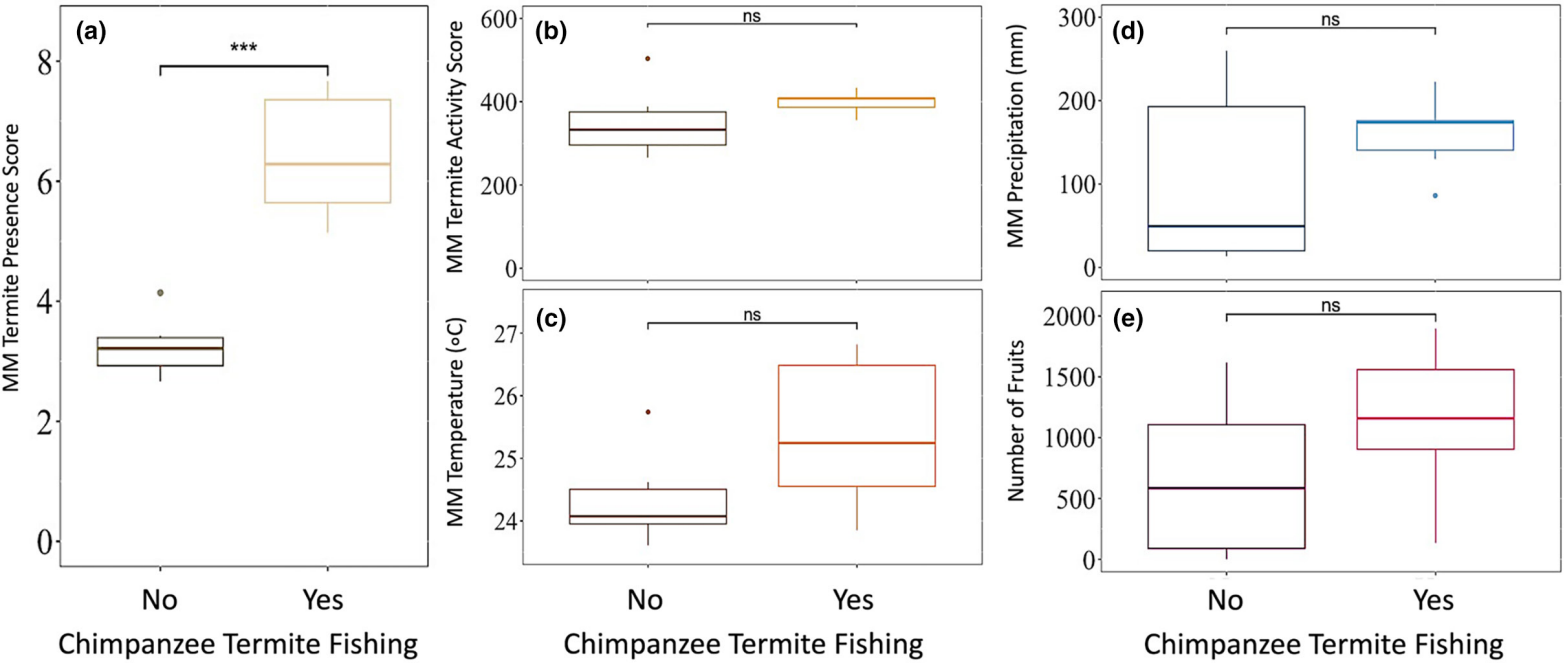
Ecological correlates of chimpanzee termite fishing seasonality. These figures show the differences in mean monthly termite presence in the soil surface (a), mean monthly termite foraging activity (b), mean monthly temperature (c), mean monthly precipitation (d), and mean monthly availability of preferred fruits (e) between months when chimpanzees have and have not been known to participate in termite fishing. MM in the figure axes denotes “mean monthly.” Termite presence in the soil surface was significantly higher during periods when chimpanzees would participate in termite fishing compared to periods when they did not (*t* = −6.569, df = 7.245, *p* < .001). All other ecological variables were not significantly different relative to when chimpanzees fished for termites (termite foraging activity: *t* = −1.259, df = 6.318, *p* = .252; mean monthly temperatures: *t* = −1.781, df = 8.315, *p* = .111; mean monthly precipitation: *t* = −1.135, df = 6.668, *p* = .295; mean preferred fruit availability: *t* = −1.259, df = 9.921, *p* = .237). Significance levels represent the following: *p < .05, **p < .01, ***p < .001, ns = non‐significant.

Termite presence in the soil surface was the only ecological variable that was significantly higher during periods when chimpanzees would participate in termite fishing compared to periods when they did not (*t* = −6.569, df = 7.245, *p* < .001) (Figure 11a). All other ecological variables were not significantly different relative to when chimpanzees fished for termites (termite foraging activity: *t* = −1.259, df = 6.318, *p* = .252; mean monthly temperatures: *t* = −1.781, df = 8.315, *p* = .111; mean monthly precipitation: *t* = −1.135, df = 6.668, *p* = .295; mean preferred fruit availability: *t* = −1.259, df = 9.921, *p* = .237) (Figure 11b–e).

#### 3.1.6 Ecological drivers of termite behavior

**FIGURE 12:**
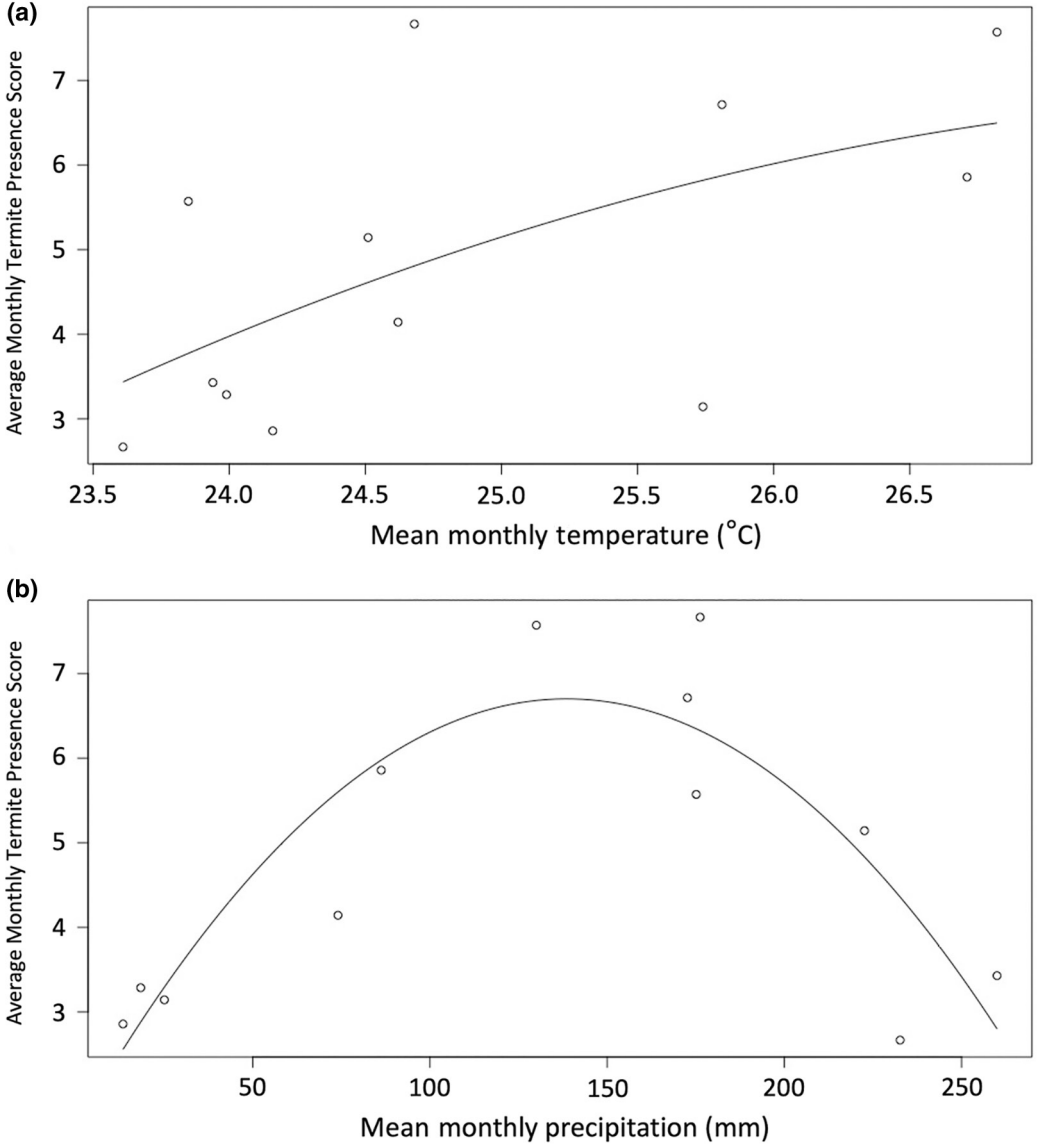
Ecological correlates of termite presence in the soil surface. These figures show the relationship between mean monthly termite presence score and mean monthly temperature (a) and mean monthly rainfall (b). The relationship between mean monthly rainfall and mean monthly termite presence score showed a much greater correlation compared to that with mean monthly temperature (mean monthly temperature: *R* ^2^ = .270; mean monthly rainfall: *R* ^2^ = .775). This suggests that rainfall is a very strong predictor of termite presence in the soil surface.

We used multiple regression to determine if either temperature or rainfall was significantly associated with termite presence. The relationship between mean monthly rainfall and termite presence was fit best with a quadratic regression (*F* = 13.9, *p* = .002, *R* ^2^ = .775) while the relationship between mean monthly temperature and termite presence was fit best with a logarithmic regression (*F* = 5.059, *p* = .048, *R* ^2^ = .270) (Figure 12). Multiple regression demonstrated that mean monthly rainfall was the primary predictor of mean monthly termite presence in the soil surface (*F* = 10.4, *p* = .004, *R* ^2^ = .796) (Table 1). Termites were most frequently present during periods with intermediate levels of mean monthly rainfall (Figure 12b).

**TABLE 1:** Multiple regression model examining the effects of mean monthly temperature and mean monthly rainfall on mean monthly termite presence.

Variable	Estimate	SE	*t*‐Value	*p*‐value
Intercept	−32.950	27.740	−1.201	.264
Log(Mean Temp)	10.850	8.608	1.260	.243
Mean Rainfall	0.062	0.016	3.840	.005**
(Mean Rainfall)^2^	−2.15e‐4	6.41e‐5	−3.361	.010**

*Note:* Significance values represent the following: *p < .05, **p < .01, ***p < .001.

## 4 DISCUSSION

Across their distribution, chimpanzees frequently consume termites, suggesting they fulfill a potentially important dietary role (Boesch et al., 2020; Bogart & Pruetz, 2011; McGrew et al., 1979; McGrew & Collins, 1985; O'Malley & Power, 2012, 2014; Sanz & Morgan, 2013). Seasonality of termite exploitation therefore poses an interesting question regarding what factors influence when chimpanzees consume this putatively valuable resource, as some combination of ecological, biological, and/or social factors may be responsible for shaping seasonality of this chimpanzee behavior. From an ecological perspective, this seasonality could represent the seasonal availability of termites, which would support the “opportunity” hypothesis of tool‐use behavior, or termites may be eaten more frequently when fruit is not available, indicating that termites are serving as fallback food items in line with the “necessity” hypothesis of tool‐use behavior (Fox et al., 1999). In the absence of an ecological explanation for termite fishing seasonality in this community, it may be that this pattern of termite consumption is instead driven by sociocultural and/or physiological factors (e.g., micronutrients from termites may only be relevant for certain chimpanzees during certain parts of the year, or may be acquired in such large quantities during this period that they need not be consumed during other parts of the year). Our study focused on whether seasonal termite fishing by Nigeria‐Cameroon chimpanzees (*P. t. ellioti*) in central Cameroon was consistent with the “necessity” and/or “opportunity” hypotheses for tool‐use behavior. Our findings revealed a relationship between rainfall, termite accessibility, and termite consumption by chimpanzees that is consistent with the “opportunity hypothesis” and with recent findings from a chimpanzee community in east Africa (Phillips et al., 2023).

### 4.1 Termite fishing seasonality in Mbam & Djerem National Park

In Mbam & Djerem National Park (MDNP), termite fishing by *P. t. ellioti* aligns with increased termite presence in the soil surface without corresponding changes to termite foraging activity, soil characteristics, or fruit availability. The water content of the termite mound soil did vary throughout the year in expected patterns given seasonal rainfall, but it was no different on average between months when chimpanzee fishing has and has not been observed. Interestingly, median water content was lower during the big rainy season period (Aug: 16% of sample, Oct: 17.4% of sample) than during the small rainy season, which may suggest some aspect of termite behavior and/or mound architecture may contribute to water‐shedding or impact soil drainage (Wood, 1988). Altogether, this evidence suggests that seasonal termite behavior is driving increased accessibility by chimpanzees. Anecdotal evidence from our camera trap videos supports recent research which finds that this seasonal accessibility is tied to termite reproductive behavior when winged alates perform reproductive flights (Phillips et al., 2023; T. Andres‐Bray, unpublished).

### 4.2 Ecological drivers of termite behavior

Previous work examining seasonal termite reproductive behavior for mound‐building termites suggests they cue mating flights based on environmental signals. Mating flights for *Macrotermes* species, a well‐known target of chimpanzee termite fishing, have been shown to be triggered by rainfall, which aligns with the findings of this study (Mitchell, 2008; Phillips et al., 2023). Other research has also identified temperature, wind speed, and humidity as important factors determining the timing of termite reproductive behavior (Mitchell, 2008). One study found that while greater rainfall was associated with larger mating flight group sizes in *Macrotermes natalensis*, they will not fly during periods of rain. Instead, mating flights in this species are correlated with the moderate‐to‐high levels of relative humidity that follow periods of high rainfall (Mitchell, 2008). This aligns with conditions in MDNP during the small rainy season, where temperatures are high and rainfall begins increasing, which could create humid conditions that precede termite mating flights. Thus, our findings suggest that as rainfall increases during this period, members of the termite colony appear to move closer to the surface of their mounds, increasing abundance in flight holes, where the defensive behavior of termite soldiers is exploited by chimpanzees during termite fishing (Nishida et al., 1999; Phillips et al., 2023).

Like Phillips et al. (2023), we identified strong relationships between rainfall and the accessibility of termites on the soil surface, with peak accessibility at intermediate rainfall levels. Our results demonstrate that chimpanzees are exploiting this pattern and timing their periods of termite fishing to align with seasonal termite accessibility. Videos from MDNP show that chimpanzees begin visiting termite mounds at MDNP more frequently as the rains begin and will perform investigative behaviors like ground‐scratching and tool probes, suggesting an awareness of the factors that influence termite accessibility, but more research is needed to explore this pattern fully (T. Andres‐Bray, unpublished). However, while *P. t. schweinfurthii* at Issa Valley prey primarily upon *Macrotermes sybhyalinus* termites (Phillips et al., 2023), evidence suggests that *P. t. ellioti* in MDNP are likely consuming *S. sphaerothorax*, a mound‐building termite often grouped within the Macrotermitinae family (Darlington, 2021). This could contribute to community‐level differences in the way tools are used, timing of termite fishing, and reliance on termites as a dietary resource. For example, video evidence from camera traps suggests that chimpanzees in MDNP do not consume alates, as alate flights in this region are occurring exclusively at night (T. Andres‐Bray, unpublished), while chimpanzees in Gombe are known to eat alates by hand when they fly out while chimpanzees are present at the termite mounds (E. Lonsdorf, personal communication). In *Macrotermes*, alates have been shown to have low amounts of dietary minerals but appear desired by chimpanzees in other communities because of a much higher fat content relative to other termite castes (O'Malley & Power, 2014). In contrast, *Macrotermes* soldiers appear to provide an array of important dietary minerals (O'Malley & Power, 2014). If this pattern is similar for other members of Macrotermitinae like *S. sphaerothorax*, this could suggest that termite fishing in MDNP serves more as a means to acquire dietary minerals, while termite fishing in areas like Gombe where alates can be eaten may provide important levels of both mineral and caloric intake.

Given that variation in termite behavior appears to contribute to variation in chimpanzee termite fishing behavior, the uncertainty regarding the termite species being fished by chimpanzees in this community represents a limitation of this study. Based on comparative abundances of termite specimens collected in this study and known species nest characteristics, we assume the most likely species being fished by Ganga chimpanzees is *S. sphaerothorax*, but we cannot exclude the possibility that *M. calvus* inhabit at least some of the mounds used in this study. Termites belonging to the known chimpanzee prey genus *Macrotermes* were identified at several mounds in very small abundances, suggesting that they are available in this region but unlikely to be the inhabitants of the mounds being fished in this study. If chimpanzees at Ganga are predating upon several different termite species, some variation in their termite fishing behavior may be a between‐mound response to differences in termite behavior between termite species. Further research is needed to explore the relationship between chimpanzee behavioral variation and termite prey behavioral variation at this fine‐scale resolution and to untangle the potentially complicated interactions among termite communities in this region.

### 4.3 Implications for chimpanzee socioecology

The results of this study support the “opportunity hypothesis,” which posits that patterns of tool use are determined by opportunities to use those tools. If those opportunities are constrained by behaviors of termite prey, this could contribute to greater behavioral variation between chimpanzee communities based on the availability and species distribution of termites. Several chimpanzee communities ignore abundant termite species, such as *Odontotermes* spp., potentially due to taste, size, or accessibility (Lesnik, 2014; Pascual‐Garrido & Scheffrahn, 2020; Sanz et al., 2014). Elements like seasonal termite reproductive behavior, which can increase prey accessibility, thus contributing to the emergence and maintenance of termite fishing within a chimpanzee community.

The results of this study did not align with the “necessity hypothesis.” Periods of chimpanzee termite fishing were not associated with the availability of preferred chimpanzee fruits, which is highest during the big rainy season but remain abundant during the small rainy season when termites are consumed. This is in line with findings from other chimpanzee communities (McGrew et al., 1979; Sanz & Morgan, 2013). Lack of support for this hypothesis in several chimpanzee communities could indicate that past ecological conditions played a more important role in current observed patterns of tool use in chimpanzees (Sanz & Morgan, 2013). Further, what is considered “necessity” may be contextual. The micronutrients available in insects, such as vitamins and minerals, may be absent or diminished in preferred chimpanzee food items (Deblauwe & Janssens, 2008; O'Malley & Power, 2012). It may be that termites provide specific, valuable nutrients, and other insect prey may serve as fallback foods when termites are not accessible.

Studies of tool use in New Caledonian crows have suggested the “relative profitability hypothesis,” which suggests that extractive tool use foraging strategies will emerge when they are more energetically profitable than nontool use foraging strategies, as embedded food items can have high nutritional value (e.g., nuts) or specific nutrients not found in preferred diet items (e.g., social insects) (Rutz et al., 2010; Rutz & St Clair, 2012). Social insects appear to be a significant source of protein and micronutrients, iron, and manganese, and may provide more biologically useful amino acids relative to plants (Barker et al., 1998; Hladik, 1977; O'Malley & Power, 2014). Chimpanzees can also easily achieve their estimated vitamin B12 requirements with termites alone (Deblauwe & Janssens, 2008). Furthermore, chimpanzee communities that have high instances of hunting vertebrates (e.g., Ngogo and Budongo in Uganda) have infrequent or complete lack of insect‐directed tool use, suggesting that social insects can serve as a more energetically profitable source of animal material for chimpanzee diets as social insects can often be found in stable locations and eaten in large amounts (Sherrow, 2005; Whiten & Boesch, 2001). Termite mound soil may also serve as a non‐negligible source of iron and aluminum (Reynolds et al., 2019). Recent work in several communities of *P. t. schweinfurthii* in East Africa found that termite mound soil contains higher levels of these two minerals compared to other chimpanzee diet items (Reynolds et al., 2019). While we did not observe any instances of direct geophagy by chimpanzees in MDNP, soil may be eaten incidentally during termite fishing, thus adding to the potential nutritional value of termite fishing. It is possible that the relative profitability of micronutrients/minerals available from termite fishing is driving this behavior by *P. t. ellioti* in MDNP. If this is the case, this further suggests that termites represent a valuable but seasonally inaccessible dietary resource for this chimpanzee community, in which case *P. t. ellioti* in MDNP may attempt to supplement their diets in other parts of the year to make up for a deficit. However, addressing this hypothesis requires detailed information on the quality, quantity, and breadth of diet items which can be difficult to acquire, particularly in nonhabituated chimpanzee communities.

The connection between climate, termite reproduction, and chimpanzee termite fishing reflects a delicate ecological balance that is vulnerable to climate change. Previous research predicts that *P. t. ellioti* inhabiting central Cameroon will experience a drastic reduction in suitable habitats in the next several decades (Sesink Clee et al., 2015). Climate change is also predicted to lead to delayed wet seasons and more intense rainfall in West Africa, which can alter termite reproductive ecology given that the timing and abundance of termite alate mating flights are strongly tied to rainfall (Dunning et al., 2018; Phillips et al., 2023). Disruption of this seasonal termite reproductive behavior could then lead to shifts in termite accessibility, which would impact chimpanzee diet, technical behaviors, and health.

## AUTHOR CONTRIBUTIONS

**Tyler C. Andres‐Bray:** Conceptualization (lead); data curation (lead); formal analysis (lead); investigation (supporting); methodology (lead); visualization (lead); writing – original draft (lead); writing – review and editing (equal). **Jeffrey Smith:** Data curation (supporting); formal analysis (supporting); writing – review and editing (supporting). **Ian Nichols:** Conceptualization (supporting); investigation (supporting); methodology (supporting); writing – review and editing (supporting). **Ekwoge E. Abwe:** Funding acquisition (equal); investigation (lead); project administration (equal); writing – review and editing (supporting). **Mary Katherine Gonder:** Conceptualization (supporting); funding acquisition (equal); investigation (supporting); methodology (supporting); project administration (equal); writing – review and editing (equal).

## CONFLICT OF INTEREST STATEMENT

The authors declare that they have no known competing financial interests, personal relationships, or other involvements that could have influenced or raised questions of bias in the work reported in this paper.

## Supporting information


Appendix S1

## Data Availability

The data that support the findings of this study are freely available at https://doi.org/10.5061/dryad.n5tb2rc3t and are included in Appendix S1 of this article for ease of access.

## References

[ece370080-bib-0001] Abwe, E. (2018). Linking behavioral diversity with genetic and ecological variation in the Nigeria‐Cameroon chimpanzee . Dissertation, Drexel University.

[ece370080-bib-0002] Abwe, E. E. , & Morgan, B. J. (2008). The Ebo forest: Four years of preliminary research and conservation of the Nigeria‐Cameroon chimpanzee (*Pan troglodytes vellerosus*). Pan Africa News, 15(2), 26–29.

[ece370080-bib-0003] Abwe, E. E. , Morgan, B. J. , Tchiengue, B. , Kentatchime, F. , Doudja, R. , Ketchen, M. E. , Teguia, E. , Ambahe, R. , Venditti, D. M. , Mitchell, M. W. , Fosso, B. , Mounga, A. , Fotso, R. C. , & Gonder, M. K. (2019). Habitat differentiation among three Nigeria–Cameroon chimpanzee (*Pan troglodytes ellioti*) populations. Ecology and Evolution, 9(3), 1489–1500. 10.1002/ece3.4871 30805176 PMC6374666

[ece370080-bib-0004] Barker, D. , Fitzpatrick, M. P. , & Dierenfeld, E. S. (1998). Nutrient composition of selected whole invertebrates. Zoo Biology, 17(2), 123–134. 10.1002/(SICI)1098-2361(1998)17:2<123::AID-ZOO7>3.0.CO;2-B

[ece370080-bib-0005] Boesch, C. , Kalan, A. K. , Mundry, R. , Arandjelovic, M. , Pika, S. , Dieguez, P. , Ayimisin, E. A. , Barciela, A. , Coupland, C. , Egbe, V. E. , Eno‐Nku, M. , Michael Fay, J. , Fine, D. , Adriana Hernandez‐Aguilar, R. , Hermans, V. , Kadam, P. , Kambi, M. , Llana, M. , Maretti, G. , … Kühl, H. S. (2020). Chimpanzee ethnography reveals unexpected cultural diversity. Nature Human Behaviour, 4(9), 910–916. 10.1038/s41562-020-0890-1 32451479

[ece370080-bib-0006] Bogart, S. L. , & Pruetz, J. D. (2011). Insectivory of savanna chimpanzees (*Pan troglodytes verus*) at Fongoli, Senegal. American Journal of Physical Anthropology, 145(1), 11–20. 10.1002/ajpa.21452 21484757

[ece370080-bib-0008] Darlington, J. P. E. C. (2021). Fungus‐growing termites (Macrotermitinae). In C. K. Starr (Ed.), Encyclopedia of social insects. Springer.

[ece370080-bib-0009] Davies, A. B. , Eggleton, P. , van Rensburg, B. J. , & Parr, C. L. (2015). Seasonal activity patterns of African savanna termites vary across a rainfall gradient. Insectes Sociaux, 62, 157–165. 10.1007/s00040-014-0386-y

[ece370080-bib-0010] Deblauwe, I. (2009). Temporal variation in insect‐eating by chimpanzees and gorillas in southeast Cameroon: Extension of niche differentiation. International Journal of Primatology, 30, 229–252. 10.1007/s10764-009-9337-2

[ece370080-bib-0011] Deblauwe, I. , & Janssens, G. P. (2008). New insights in insect prey choice by chimpanzees and gorillas in southeast Cameroon: The role of nutritional value. American Journal of Physical Anthropology, 135(1), 42–55. 10.1002/ajpa.20703 17902166

[ece370080-bib-0012] Dunning, C. M. , Black, E. , & Allan, R. P. (2018). Later wet seasons with more intense rainfall over Africa under future climate change. Journal of Climate, 31(23), 9719–9738. 10.1175/JCLI-D-18-0102.1

[ece370080-bib-0013] Dutton, P. , & Chapman, H. (2015). Dietary preferences of a submontane population of the rare Nigerian‐Cameroon chimpanzee (*Pan troglodytes ellioti*) in Ngel Nyaki Forest reserve, Nigeria. American Journal of Primatology, 77(1), 86–97. 10.1002/ajp.22313 25231641

[ece370080-bib-0014] Fox, E. A. , Sitompul, A. F. , & Van Schaik, C. P. (1999). Intelligent tool use in wild Sumatran orangutans. The Mentality of Gorillas and Orangutans, 480, 99–116.

[ece370080-bib-0015] Furuichi, T. , Hashimoto, C. , & Tashiro, Y. (2001). Extended application of a marked‐nest census method to examine seasonal changes in habitat use by chimpanzees. International Journal of Primatology, 22, 913–928. 10.1023/A:1012057403512

[ece370080-bib-0016] Goodall, J. (1964). Tool‐using and aimed throwing in a community of free‐living chimpanzees. Nature, 201(4926), 1264–1266. 10.1038/2011264a0 14151401

[ece370080-bib-0017] Hanusz, Z. , Tarasinska, J. , & Zielinski, W. (2016). Shapiro–Wilk test with known mean. REVSTAT‐Statistical Journal, 14(1), 89–100. 10.57805/revstat.v14i1.180

[ece370080-bib-0018] Hernandez‐Aguilar, R. A. , Moore, J. , & Pickering, T. R. (2007). Savanna chimpanzees use tools to harvest the underground storage organs of plants. Proceedings of the National Academy of Sciences of the United States of AMERICA, 104(49), 19210–19213. 10.1073/pnas.0707929104 18032604 PMC2148269

[ece370080-bib-0019] Hladik, C. M. (1977). Chimpanzees of Gabon and chimpanzees of Gombe: Some comparative data on the diet. In T. H. Clutton‐Brock (Ed.), Primate ecology: Studies of feeding and ranging behaviour in lemurs, monkeys, and apes (pp. 81–501). Academic Press.

[ece370080-bib-0020] Isbell, L. A. (1998). Diet for a small primate: Insectivory and gummivory in the (large) patas monkey (*Erythrocebus patas pyrrhonotus*). American Journal of Primatology, 45(4), 381–398. 10.1002/(SICI)1098-2345(1998)45:4<381::AID-AJP5>3.0.CO;2-S 9702283

[ece370080-bib-0021] Kamgang, S. A. , Bobo, K. S. , Maisels, F. , Ambahe, R. D. D. , Ambassa Ongono, D. E. , Gonder, M. K. , Johnson, P. , Marino, J. , & Sinsin, B. (2018). The relationship between the abundance of the Nigeria‐Cameroon chimpanzee (*Pan troglodytes ellioti*) and its habitat: A conservation concern in Mbam‐Djerem National Park, Cameroon. BMC Ecology, 18, 1–14. 10.1186/s12898-018-0199-3 30285707 PMC6167774

[ece370080-bib-0022] Kay, R. F. (1975). The functional adaptations of primate molar teeth. American Journal of Physical Anthropology, 43(2), 195–215. 10.1002/ajpa.1330430207 810034

[ece370080-bib-0024] Lesnik, J. J. (2014). Termites in the hominin diet: A meta‐analysis of termite genera, species and castes as a dietary supplement for South African robust australopithecines. Journal of Human Evolution, 71, 94–104. 10.1016/j.jhevol.2013.07.015 24613098

[ece370080-bib-0025] Lonsdorf, E. V. , Eberly, L. E. , & Pusey, A. E. (2004). Sex differences in learning in chimpanzees. Nature, 428(6984), 715–716. 10.1038/428715a 15085121

[ece370080-bib-0026] MacKinnon, J. , & MacKinnon, K. (1980). The behavior of wild spectral tarsiers. International Journal of Primatology, 1, 361–379. 10.1007/BF02692280

[ece370080-bib-0027] Maisels, F. (2005). Mbam Djerem National Park, Cameroon: at the forest's edge. Canopee, 27, 2–6.

[ece370080-bib-0028] McGrew, W. C. (2014). The ‘other faunivory’ revisited: Insectivory in human and non‐human primates and the evolution of human diet. Journal of Human Evolution, 71, 4–11. 10.1016/j.jhevol.2013.07.016 24560030

[ece370080-bib-0029] McGrew, W. C. , & Collins, D. A. (1985). Tool use by wild chimpanzees (Pan troglodytes) to obtain termites (*Macrotermes herus*) in the Mahale Mountains, Tanzania. American Journal of Primatology, 9(1), 47–62. 10.1002/ajp.1350090106 31986794

[ece370080-bib-0030] McGrew, W. C. , Tutin, C. E. , & Baldwin, P. J. (1979). Chimpanzees, tools, and termites: Cross‐cultural comparisons of Senegal, Tanzania, and Rio Muni. Man, 14, 185–214. 10.2307/2801563

[ece370080-bib-0031] Mitchell, J. D. (2008). Swarming flights of the fungus‐growing termite, *Macrotermes natalensis* (Haviland) (Isoptera: Macrotermitinae), and the environmental factors affecting their timing and duration. African Entomology, 16(2), 143–152.

[ece370080-bib-0032] Mitchell, M. W. , Locatelli, S. , Ghobrial, L. , Pokempner, A. A. , Sesink Clee, P. R. , Abwe, E. E. , Nicholas, A. , Nkembi, L. , Anthony, N. M. , Morgan, B. J. , Fotso, R. , Peeters, M. , Hahn, B. H. , & Gonder, M. K. (2015). The population genetics of wild chimpanzees in Cameroon and Nigeria suggests a positive role for selection in the evolution of chimpanzee subspecies. BMC Evolutionary Biology, 15, 1–15. 10.1186/s12862-014-0276-y 25608610 PMC4314757

[ece370080-bib-0033] Nishida, T. , & Hiraiwa, M. (1982). Natural history of a tool‐using behavior by wild chimpanzees in feeding upon wood‐boring ants. Journal of Human Evolution, 11(1), 73–99. 10.1016/S0047-2484(82)80033-X

[ece370080-bib-0034] Nishida, T. , Kano, T. , Goodall, J. , McGrew, W. C. , & Nakamura, M. (1999). Ethogram and ethnography of Mahale chimpanzees. Anthropological Science, 107(2), 141–188. 10.1537/ase.107.141

[ece370080-bib-0036] Oates, J. F. , Doumbe, O. , Dunn, A. , Gonder, M. K. , Ikemeh, R. , Imong, I. , Morgan, B. J. , Ogunjemite, B. , & Sommer, V. (2016). Pan troglodytes *ssp*. ellioti. *The IUCN Red List of Threatened Species 2016*: e.T40014A17990330. 10.2305/IUCN.UK

[ece370080-bib-0037] O'Malley, R. C. , & Power, M. L. (2012). Nutritional composition of actual and potential insect prey for the Kasekela chimpanzees of Gombe National Park, Tanzania. American Journal of Physical Anthropology, 149(4), 493–503. 10.1002/ajpa.22151 23115107

[ece370080-bib-0038] O'Malley, R. C. , & Power, M. L. (2014). The energetic and nutritional yields from insectivory for Kasekela chimpanzees. Journal of Human Evolution, 71, 46–58. 10.1016/j.jhevol.2013.09.014 24698197

[ece370080-bib-0039] Pascual‐Garrido, A. , & Scheffrahn, R. H. (2020). Cultural dietary stasis? Four decades on, Mahale chimpanzees still favour Macrotermes. Pan Africa News, 27(1), 6–9.

[ece370080-bib-0040] Phillips, S. , Piel, A. K. , Stewart, F. A. , & Oelze, V. M. (2023). A chimpanzee's time to feast: Seasonality of Macrotermes flight hole activity and alate dispersal flights detected by termite‐fishing experiments and camera traps in the Issa Valley, Tanzania. Frontiers in Ecology and Evolution, 11, 1289433. 10.3389/fevo.2023.1289433

[ece370080-bib-0041] Raubenheimer, D. , & Rothman, J. M. (2013). Nutritional ecology of entomophagy in humans and other primates. Annual Review of Entomology, 58, 141–160. 10.1146/annurev-ento-120710-100713 23039342

[ece370080-bib-0202] Reynolds, V. , Pascual‐Garrido, A. , Lloyd, A. W. , Lyons, P. , & Hobaiter, C. (2019). Possible mineral contributions to the diet and health of wild chimpanzees in three East African forests. American Journal of Primatology, 81(6), 141–160. 10.1002/ajp.22978 31090097

[ece370080-bib-0042] Rutz, C. , Bluff, L. A. , Reed, N. , Troscianko, J. , Newton, J. , Inger, R. , Kacelnik, A. , & Bearhop, S. (2010). The ecological significance of tool use in New Caledonian crows. Science, 329(5998), 1523–1526. 10.1126/science.1192053 20847272

[ece370080-bib-0043] Rutz, C. , & St Clair, J. J. (2012). The evolutionary origins and ecological context of tool use in New Caledonian crows. Behavioural Processes, 89(2), 153–165. 10.1016/j.beproc.2011.11.005 22209954

[ece370080-bib-0044] Sanz, C. M. , Deblauwe, I. , Tagg, N. , & Morgan, D. B. (2014). Insect prey characteristics affecting regional variation in chimpanzee tool use. Journal of Human Evolution, 71, 28–37. 10.1016/j.jhevol.2013.07.017 24602365

[ece370080-bib-0045] Sanz, C. M. , & Morgan, D. B. (2013). Ecological and social correlates of chimpanzee tool use. Philosophical Transactions of the Royal Society B: Biological Sciences, 368(1630), 20120416. 10.1098/rstb.2012.0416 PMC4027411 24101626

[ece370080-bib-0046] Sesink Clee, P. R. , Abwe, E. E. , Ambahe, R. D. , Anthony, N. M. , Fotso, R. , Locatelli, S. , Maisels, F. , Mitchell, M. W. , Morgan, B. J. , Pokempner, A. A. , & Gonder, M. K. (2015). Chimpanzee population structure in Cameroon and Nigeria is associated with habitat variation that may be lost under climate change. BMC Evolutionary Biology, 15(1), 1–13. 10.1186/s12862-014-0275-z 25608567 PMC4314735

[ece370080-bib-0201] Sherrow, H. M. (2005). Tool use in insect foraging by the chimpanzees of Ngogo, Kibale National Park, Uganda. American Journal of Primatology, 65(4), 377–383. 10.1002/ajp.20122 15834891

[ece370080-bib-0047] Smith, T. B. , Wayne, R. K. , Girman, D. J. , & Bruford, M. W. (1997). A role for ecotones in generating rainforest biodiversity. Science, 276, 1855–1857. 10.1126/science.276.5320.1855

[ece370080-bib-0048] Sommer, V. , Buba, U. , Jesus, G. , & Pascual‐Garrido, A. (2017). Sustained myrmecophagy in Nigerian chimpanzees: Preferred or fallback food? American Journal of Physical Anthropology, 162(2), 328–336. 10.1002/ajpa.23122 27779749

[ece370080-bib-0049] Stewart, F. A. , & Piel, A. K. (2014). Termite fishing by wild chimpanzees: New data from Ugalla, western Tanzania. Primates, 55, 35–40. 10.1007/s10329-013-0362-6 23720026

[ece370080-bib-0050] Thorne, B. L. , Collins, M. S. , & Bjorndal, K. A. (1996). Architecture and nutrient analysis of arboreal carton nests of two neotropical Nasutitermes species (Isoptera: Termitidae), with notes on embedded nodules. Florida Entomologist, 79, 27.

[ece370080-bib-0051] Whiten, A. (2017). A second inheritance system: The extension of biology through culture. Interface Focus, 7(5), 20160142. 10.1098/rsfs.2016.0142 28839918 PMC5566806

[ece370080-bib-0052] Whiten, A. , & Boesch, C. (2001). The cultures of chimpanzees. Scientific American, 284(1), 60–67. 10.1038/scientificamerican0101-60 11132425

[ece370080-bib-0053] Whiten, A. , Goodall, J. , McGrew, W. C. , Nishida, T. , Reynolds, V. , Sugiyama, Y. , Tutin, C. E. , Wrangham, R. W. , & Boesch, C. (1999). Cultures in chimpanzees. Nature, 399(6737), 682–685. 10.1038/21415 10385119

[ece370080-bib-0054] Wood, T. G. (1988). Termites and the soil environment. Biology and Fertility of Soils, 6, 228–236. 10.1007/BF00260819

[ece370080-bib-0055] Woon, J. S. , Boyle, M. J. W. , Ewers, R. M. , Chung, A. , & Eggleton, P. (2019). Termite environmental tolerances are more linked to desiccation than temperature in modified tropical forests. Insectes Sociaux, 66, 57–64. 10.1007/s00040-018-0664-1

[ece370080-bib-0056] World Bank Group . (2020). Climate change knowledge portal for development practitioners and policy makers . https://climateknowledgeportal.worldbank.org

