# Supplementary material for: Ecological correlates of chimpanzee termite fishing behavior in Mbam & Djerem National Park, Cameroon
Source: Ecol Evol. 2024 Jul 24;14(7):e70080. doi: 10.1002/ece3.70080 (PMC11268952; doi:10.1002/ece3.70080)
Supplement: Supplementary file 1 — Appendix S1 [file ECE3-14-e70080-s001.zip › README - Data Descriptions.rtf]

Title=“Metadata descriptions for Ecology & Evolution Submission”Manuscript Title=“ECOLOGICAL CORRELATES OF CHIMPANZEE TERMITE FISHING BEHAVIOR IN MBAM & DJEREM NATIONAL PARK, CAMEROON”Creator=“Andres-Bray, Tyler”Subject=“1_SeasonalTermitePredation.xlsx”Description=“Count data of the number of videos captured on camera traps during this study that included large-bodied mammalian termite predators.”Data Columns=“Year: Year video was recorded, Month: Month video was recorded, Smutsia gigantea: Video counts for Giant Pangolin, Phataginus tricuspis: Video counts for Tree Pangolin, Orycteropus afer: Video counts for Aardvark, Civettictis civetta: Video counts for African civet, & Pan troglodytes ellioti: Video counts for Nigeria-Cameroon Chimpanzee.Date=“Dec-2023”Type=“Text”Format=“application/Excel”Location=“Uploaded as Supplemental Material”Language=“en”Subject=“2_TermiteSurveyData.xlsx”Description=“All data from termite survey to monitor termite presence in the top 6 inches of the soil surface and foraging activity. Presence in soil surface was determined using active searching in 5 1mx1m quadrats tangent to each of the 7 termite mounds monitored. Presence in each quadrat was assessed using a 0-5 scale for speed and efficiency, where 0: no termites, 1: 1-25 termites, 2: 26-50 termites, 3: 51-75 termites, 4: 76-100 termites, and 5: 100+ termites. Foraging activity was assessed using 10 toilet paper cellulose baits at each mound, 5 secured above ground and 5 secured below ground. Baits were assessed monthly for termite foraging activity using a 0-5 scale, where 0: no damage, 1: 1-25% of bait eaten, 2: 26-50% of bait eaten, 3: 51-75% of bait eaten, 4: 76-99% of bait eaten, and 5: bait completely consumed. During bait collection, termites and non-termite arthropods present on the baits at time of collection were noted in order to calculate the assumed degree of bait score that can reliably be attributed to termite activity. Missing data is explained in the notes, and the foraging activity data is offset by one month due to the nature of the survey, as baits were set up in April 2022 and could not be assessed until May 2022.”Data Columns=”Mound: Unique letter code for each termite mound, Mtype: type of termite mound - AE: aerial/epigeal and UN: underground/subterranean, Transect: Unique number code for location of mound - this field site has 10 2-km transects numbered from nearest to furthest from camp, Meter: Length in meters where termite mound is found along the designated transect, Date: Date of data collection, Time: time of data collection, Month: Month of data collection, Q1-Q5: Quadrats 1 through 5 - denoting the average termite presence score for each quadrat at time of data collection, Qavg: Mean presence score across all five quadrats at time of data collection, Qmed: Median presence score across all five quadrats at time of data collection, Qran: Range of presence scores across all five quadrats at time of data collection, Qtot: Summation of presence scores across all five quadrats at time of data collection, Presence Notes: Relevant information about termite presence data collection at the mound in question, HS1-5: Hors-sol 1 through 5 - refers to above-ground cellulose baits at each mound and denotes the foraging activity score for each bait at time of data collection, HS#Term: Refers to the the number of termites found in each above ground bait during data collection, HS#Arth: Refers to the the number of non-termite arthropods found in each above ground bait during data collection, HSavg: Mean termite foraging activity score across all five above ground baits at time of data collection, HSmed: Median termite foraging activity score across all five above ground baits at time of data collection, HSran: Range of termite foraging activity scores across all five above ground baits at time of data collection, HStot: Summation of termite foraging activity scores across all five above ground at time of data collection, ST1-5: Souterrain 1 through 5 - refers to below-ground cellulose baits at each mound and denotes the foraging activity score for each bait at time of data collection, ST#Term: Refers to the the number of termites found in each below ground bait during data collection, ST#Arth: Refers to the the number of non-termite arthropods found in each below ground bait during data collection, STavg: Mean termite foraging activity score across all five below ground baits at time of data collection, STmed: Median termite foraging activity score across all five below ground baits at time of data collection, STran: Range of termite foraging activity scores across all five below ground baits at time of data collection, STtot: Summation of termite foraging activity scores across all five below ground at time of data collection, Activity Notes: Relevant information about foraging activity data collection at the mound in question - denotes that data collection on foraging activity did not occur in the first month when initial termite presence data was collected as the baits needed to be set up for one month before foraging activity could be assess - also denotes when scores were not given to certain baits based on evidence of disruption by mammals.”Date=“May-2023”Type=“Text”Format=“application/Excel”Location=“Uploaded as Supplemental Material”Language=“en”Subject=“3_ClimateData.xlsx”Description=“Average temperature and rainfall data from 1991-2020 for Adamoua, the state in Cameroon where this study took place. These data are courtesy of the Climate Change Knowledge Portal which incorporates historical data from Climatic Research Unit of University of East Anglia.”Data Columns=”Category: Month of the year, Avg Min Temp: Average minimum surface air temperature per month for the region recorded in degrees Celsius, Avg Mean Temp: Average mean surface air temperature per month for the region recorded in degrees Celsius, Avg Max Temp: Average maximum surface air temperature per month for the region recorded in degrees Celsius, Precipitation: Average rainfall per month measured in millimeters.”Date=“Mar-2023”Type=“Text”Format=“application/Excel”Location=“Uploaded as Supplemental Material”URL: “https://climateknowledgeportal.worldbank.org/country/cameroon/climate-data-historical”Language=“en”Subject=“4_FruitfallData.xlsx”Description=“Biomonitoring survey providing counts, locations, and species information for all fruits identified along the 10 2-km transects near Ganga Research Station from January 2016 - September 2017 and from January 2019 - December 2021.”Data Columns=“Date: Date of data collection, Study Area: Ganga Research Station, where this biomonitoring survey took place, Fruit count: Number of each identified fruit found, Feeding sign: Provides information on the presence or absence of evidence of chimpanzee feeding - Yes indicates there is evidence of chimpanzee feeding and None means there was not, Fruit Species: Taxonomic identification of fruit to the lowest taxonomic level possible - often species but occasionally genus when specific species information could not be obtained e.g., Ficus spp. Unknown fruit species were photographed with their seeds and assigned numbers so that they could still be identified in macroscopic fecal diet analysis, Transect: Unique number code for location of mound - this field site has 10 2-km transects numbered from nearest to furthest from camp, Meter: Length in meters where termite mound is found along the designated transect.”Date=“August-2022”Type=“Text”Format=“application/Excel”Location=“Uploaded as Supplemental Material”Language=“en”Subject=“5_SoilData.xlsx”Description=“Soil pH, soil weight, and water content characteristics of soil taken from each mound. Two soil samples were collected every two months from each mound, one from the summit/center of the mound and one from the base/edge of the mound.”Data Columns=”Mound: Unique letter code for each termite mound, Mtype: type of termite mound - AE: aerial/epigeal and UN: underground/subterranean, Transect: Unique number code for location of mound - this field site has 10 2-km transects numbered from nearest to furthest from camp, Meter: Length in meters where termite mound is found along the designated transect, Date: Date of data collection, Sample Type: location of collection of the sample - S: Summit, B: Base, Month: Month of data collection, pH: pH value of each sample - pH was calculated by mixing a uniform amount of each sample with water in a 2:5 ratio of soil to water and using a pH meter to determine pH levels, Initial Soil Weight (g): The initial weight of the remaining soil sample in grams using a digital milligram scale, Dry Soil Weight (g): The weight of the remaining soil sample in grams after drying with silica, Water Weight (g): Calculated as Initial Soil Weight - Dry Soil Weight for each soil sample - represents the amount in grams of the initial weight that was due to water in the soil, Water Content: Calculated as Water Weight/Initial Soil Weight - represents the proportion of each soil sample that was water.”Date=“May-2023”Type=“Text”Format=“application/Excel”Location=“Uploaded as Supplemental Material”Language=“en”Subject=“6_DataForAnalysis.xlsx”Description=“Data compiled from previously described sources to represent average monthly values of termite behavior, climate, and fruit availability for this study. These data were used to perform the bulk of the statistical analyses described in this manuscript.”Data Columns=”Month: Month of the year, Season: Corresponding season during each month in this region - this region experiences broadly three distinct seasons determined by rainfall: a dry season from November to February, a little rainy season from March to June, and a big rainy season from July to October, Fishing.Bin: Binary variable denoting whether chimpanzee termite fishing has ever been observed during this month - assessed from camera trap videos, Tpres: the average of the QTot scores from 2_TermiteSurveyData.xlsx across all termite mounds for each month - summed presence scores for each mound were used as the summation of ordinal values helps approximate continuous values and the normal distribution of these data were confirmed during analysis, Tact: average transformed termite foraging activity scores per month - the summed value of HSTot and STTot scores from 2_TermiteSurveyData.xlsx were transformed using the regression equation calculated from bait scores and number of termites preset in each bait in order to create a measure of bait activity that can be confidently attributed to termite activity - further description is available in the Methods section of the manuscript, MeanTemp: average monthly temperature for this region - acquired from the Avg Mean Temp column in 3_ClimateData.xlsx, MeanPrecip: average monthly rainfall for this region - acquired from the Precipitation column in 3_ClimateData.xlsx, Fruit Availability: Average number of fallen fruits per month - calculated from 4_FruitfallData.xlsx, Preferred Fruit Availability: Number of fallen fruits per month of only preferred fruit species - calculated from 4_FruitfallData.xlsx - fruit preference in this region included the top 9 most eaten fruits by chimpanzees in this region and was obtained from Abwe, 2018 which identified diet components in chimpanzees from this region using macroscopic fecal diet analysis. These fruit species include Landolphia spp., Uapaca guineensis, Ficus spp., Pseudospondias macrocarpa, Myrianthus arboreus, Grewia spp., Canarium schweinfurthii, and 3 unidentified fruit species for which we obtained seeds for reference.Date=“May-2023”Type=“Text”Format=“application/Excel”Location=“Uploaded as Supplemental Material”Reference=“Abwe, E. (2018). Linking behavioral diversity with genetic and ecological variation in the Nigeria-Cameroon chimpanzee. Dissertation: Drexel University.”Language=“en”Subject=“Ecological Correlates of Termite Fishing Seasonality.R”Description=“All R code used for statistical analysis in this study to determine seasonal differences in ecological variables listed in 6_DataForAnalysis.xlsx, differences in these ecological variables between periods when chimpanzees do and do not participate in termite fishing, and the effects of climate on termite behavior.”Date=“Jun-2023”Type=“Text”Format=“application/R”Location=“Uploaded as Supplemental Material”Language=“en”
